# Supplementary material for: Fossorial adaptations in African mole-rats (Bathyergidae) and the unique appendicular phenotype of naked mole-rats
Source: Commun Biol. 2022 Jun 1;5:526. doi: 10.1038/s42003-022-03480-z (PMC9159980; doi:10.1038/s42003-022-03480-z)
Supplement: Supplementary file 2 — Supplementary Information [file 42003_2022_3480_MOESM2_ESM.docx]

**Supplementary Information**

**Fossorial adaptations in African mole-rats (Bathyergidae) and the unique appendicular phenotype of naked mole-rats**

*Germán Montoya-Sanhueza, Gabriel Šaffa, Radim Šumbera, Anusuya Chinsamy, Jennifer U. M. Jarvis, Nigel C. Bennett*

**Contents:**

Supplementary Tables (p. 2-28)

Supplementary Figures (p. 29)

Supplementary Methods (p. 30-31)

Supplementary References (p. 32-35)

**Abbreviations**

Deltoid tuberosity **DT**

Deltopectoral crest **DC**

Distal fusion of tibia-fibula **DFTFi**

Epicondyles (Humerus) **EH**

Fibula **Fi**

Femoral head **FH**

Humerus **H**

Humeral head **HH**

Index of fossorial ability **IFA**

Grater trochanter **GT**

Grater tubercle **GTu**

Lesser trochanter **LT**

Olecranon process **OP**

Relative position of DT **RDT**

Third trochanter **TT**

Tibia **T**

Tibio-fibular junction index **TJI**

Trochlear notch **TN**

**SUPPLEMENTARY TABLES**

**Supplementary Table 1.** Total number of bathyergid individuals analyzed in this study (*n* = 382). Data includes ID code, sex, institution of origin (IO), ontogenetic stage (OS), limb bones and bone superstructures. Outgroup specimens (*n* = 19) from Petromuridae and Hystricidae were also included. The bones analyzed are humerus (H, *n* = 380), ulna (U, *n* = 376), femur (F, *n* = 323), tibia (T, *n* = 312), and fibula (Fi, *n* = 312). Presence/absence of bone superstructures including a projected deltoid tuberosity (DT), olecranon process (OP), third trochanter (TT), and distal tibio-fibular fusion (DFTFi). Some specimens are from captivity (ca). Scanned bones are indicated with an asterisk (*). Sample size for each species: *Bathyergus suillus* (*n* = 78); *B. janetta* (*n* = 6); *Heliophobius argenteocinereus* (*n* = 38); *Georychus capensis* (*n* = 51); *Cryptomys hottentotus* (*n* = 53); *Fukomys mechowii* (*n* = 32); *Fukomys damarensis* (*n* = 48); *Heterocephalus glaber* (*n* = 76); *Hystrix africaeaustralis* (*n* = 18); *Petromus typicus* (*n* = 1). OS: newborn (0), pup (1), juvenile (2), and adult (3). Sex: males (M) and females (F). Institutions: Kalahari Mole-Rat Project, Kalahari Research Centre (KRC); Department of Biological Sciences, University of Cape Town (UCT); Mammal Research Institute, Department of Zoology and Entomology, University of Pretoria (UP); Department of Zoology, Faculty of Science, University of South Bohemia (USB); Paläontologisches Institut und Museum, Universität Zürich (UZ).

| Species | ID Code | Sex | Age | OS | IO | H | U | F | T-Fi | DT | OP | TT | DFTFi |
| --- | --- | --- | --- | --- | --- | --- | --- | --- | --- | --- | --- | --- | --- |
| *Bathyergus suillus* | 219 | F |  | 2 | UCT | H | U | F | T-Fi | Yes | Yes | Yes | Yes |
| *Bathyergus suillus* | 307 | F |  | 2 | UCT | H | U | F | T-Fi | Yes | Yes | Yes | Yes |
| *Bathyergus suillus* | 314 | F |  | 3 | UCT | H | U | F | T-Fi | Yes | Yes | Yes | Yes |
| *Bathyergus suillus* | 333 | F |  | 2 | UCT | H | U | F | T-Fi | Yes | Yes | Yes | Yes |
| *Bathyergus suillus* | 365 | F |  | 3 | UCT | H | U | F | T-Fi | Yes | Yes | Yes | Yes |
| *Bathyergus suillus* | 366 | F |  | 3 | UCT | H | U | F | T-Fi | Yes | Yes | Yes | Yes |
| *Bathyergus suillus* | 377 | F |  | 3 | UCT | H | U | F | T-Fi | Yes | Yes | Yes | Yes |
| *Bathyergus suillus* | 717 | F |  | 3 | UCT | H | U | F | T-Fi | Yes | Yes | Yes | Yes |
| *Bathyergus suillus* | 721 | F |  | 3 | UCT | H | U | F | T-Fi | Yes | Yes | Yes | Yes |
| *Bathyergus suillus* | 911 | F |  | 3 | UCT | H | U | F | T-Fi | Yes | Yes | Yes | Yes |
| *Bathyergus suillus* | 913 | F |  | 3 | UCT | H | U | F | T-Fi | Yes | Yes | Yes | Yes |
| *Bathyergus suillus* | 938 | F |  | 3 | UCT | H | U | F | T-Fi | Yes | Yes | Yes | Yes |
| *Bathyergus suillus* | 982 | F |  | 3 | UCT | H | U | F | T-Fi | Yes | Yes | Yes | Yes |
| *Bathyergus suillus* | 1085 | F |  | 3 | UCT | H | U | F | T-Fi | Yes | Yes | Yes | Yes |
| *Bathyergus suillus* | 1138 | F |  | 3 | UCT | H | U | F | T-Fi | Yes | Yes | Yes | Yes |
| *Bathyergus suillus* | 1144 | F |  | 3 | UCT | H | U | F | T-Fi | Yes | Yes | Yes | Yes |
| *Bathyergus suillus* | 1153 | F |  | 3 | UCT | H | U | F | T-Fi | Yes | Yes | Yes | Yes |
| *Bathyergus suillus* | 1155 | F |  | 3 | UCT | H | U | F | T-Fi | Yes | Yes | Yes | Yes |
| *Bathyergus suillus* | 1163 | F |  | 3 | UCT | H | U | F | T-Fi | Yes | Yes | Yes | Yes |
| *Bathyergus suillus* | 1169* | F |  | 3 | UCT | H | U | F | T-Fi | Yes | Yes | Yes | Yes |
| *Bathyergus suillus* | 1171 | F |  | 3 | UCT | H | U | F | T-Fi | Yes | Yes | Yes | Yes |
| *Bathyergus suillus* | 1332 | F |  | 3 | UCT | H | U | F | T-Fi | Yes | Yes | Yes | Yes |
| *Bathyergus suillus* | 1336 | F |  | 3 | UCT | H | U | F | T-Fi | Yes | Yes | Yes | Yes |
| *Bathyergus suillus* | 1373* | F |  | 2 | UCT | H | U | F | T-Fi | Yes | Yes | Yes | Yes |
| *Bathyergus suillus* | 217 | M |  | 3 | UCT | H | U | F | T-Fi | Yes | Yes | Yes | Yes |
| *Bathyergus suillus* | 220 | M |  | 3 | UCT | H | U | F | T-Fi | Yes | Yes | Yes | Yes |
| *Bathyergus suillus* | 223 | M |  | 2 | UCT | H | U | F | T-Fi | Yes | Yes | Yes | Yes |
| *Bathyergus suillus* | 300 | M |  | 2 | UCT | H | U | F | T-Fi | Yes | Yes | Yes | Yes |
| *Bathyergus suillus* | 313 | M |  | 3 | UCT | H | U | F | T-Fi | Yes | Yes | Yes | Yes |
| *Bathyergus suillus* | 376 | M |  | 3 | UCT | H | U | F | T-Fi | Yes | Yes | Yes | Yes |
| *Bathyergus suillus* | 713 | M |  | 3 | UCT | H | U | F | T-Fi | Yes | Yes | Yes | Yes |
| *Bathyergus suillus* | 765 | M |  | 3 | UCT | H | U | F | T-Fi | Yes | Yes | Yes | Yes |
| *Bathyergus suillus* | 861 | M |  | 3 | UCT | H | U | F | T-Fi | Yes | Yes | Yes | Yes |
| *Bathyergus suillus* | 910 | M |  | 3 | UCT | H | U | F | T-Fi | Yes | Yes | Yes | Yes |
| *Bathyergus suillus* | 964 | M |  | 3 | UCT | H | U | F | T-Fi | Yes | Yes | Yes | Yes |
| *Bathyergus suillus* | 965 | M |  | 3 | UCT | H | U | F | T-Fi | Yes | Yes | Yes | Yes |
| *Bathyergus suillus* | 1039 | M |  | 3 | UCT | H | U | F | T-Fi | Yes | Yes | Yes | Yes |
| *Bathyergus suillus* | 1050 | M |  | 3 | UCT | H | U | F | T-Fi | Yes | Yes | Yes | Yes |
| *Bathyergus suillus* | 1139 | M |  | 3 | UCT | H | U | F | T-Fi | Yes | Yes | Yes | Yes |
| *Bathyergus suillus* | 1154 | M |  | 3 | UCT | H | U | F | T-Fi | Yes | Yes | Yes | Yes |
| *Bathyergus suillus* | 1338 | M |  | 3 | UCT | H | U | F | T-Fi | Yes | Yes | Yes | Yes |
| *Bathyergus suillus* | 1339 | M |  | 3 | UCT | H | U | F | T-Fi | Yes | Yes | Yes | Yes |
| *Bathyergus suillus* | 332 | M |  | 2 | UCT | H | U | F | T-Fi | Yes | Yes | Yes | Yes |
| *Bathyergus suillus* | 337 | M |  | 2 | UCT | H | U | F | T-Fi | Yes | Yes | Yes | Yes |
| *Bathyergus suillus* | 367 | F |  | 2 | UCT | H | U | F | T-Fi | Yes | Yes | Yes | Yes |
| *Bathyergus suillus* | 866 | M |  | 2 | UCT | H | U | F | T-Fi | Yes | Yes | Yes | Yes |
| *Bathyergus suillus* | 1245 | F |  | 2 | UCT |  |  | F | T-Fi |  |  | Yes | Yes |
| *Bathyergus suillus* | 283* | M |  | 2? | UCT | H | U | F | T-Fi | Yes | Yes | Yes | Yes |
| *Bathyergus suillus* | F10 | F |  | 3 | UP | H | U |  |  | Yes | Yes |  |  |
| *Bathyergus suillus* | F2 | F |  | 3 | UP | H | U |  |  | Yes | Yes |  |  |
| *Bathyergus suillus* | F5 | F |  | 3 | UP | H | U |  |  | Yes | Yes |  |  |
| *Bathyergus suillus* | F7 | F |  | 3 | UP | H | U |  |  | Yes | Yes |  |  |
| *Bathyergus suillus* | S10* | F |  | 3 | UP | H | U |  |  | Yes | Yes |  |  |
| *Bathyergus suillus* | S11 | F |  | 3 | UP | H | U |  |  | Yes | Yes |  |  |
| *Bathyergus suillus* | S12 | F |  | 3 | UP | H | U |  |  | Yes | Yes |  |  |
| *Bathyergus suillus* | S13 | F |  | 3 | UP | H | U |  |  | Yes | Yes |  |  |
| *Bathyergus suillus* | S14 | F |  | 3 | UP | H | U |  |  | Yes | Yes |  |  |
| *Bathyergus suillus* | S16 | F |  | 3 | UP | H | U |  |  | Yes | Yes |  |  |
| *Bathyergus suillus* | S17 | F |  | 3 | UP | H | U |  |  | Yes | Yes |  |  |
| *Bathyergus suillus* | S2 | F |  | 3 | UP | H | U |  |  | Yes | Yes |  |  |
| *Bathyergus suillus* | S20 | F |  | 3 | UP | H | U |  |  | Yes | Yes |  |  |
| *Bathyergus suillus* | S3 | F |  | 3 | UP | H | U |  |  | Yes | Yes |  |  |
| *Bathyergus suillus* | S6 | F |  | 3 | UP | H | U |  |  | Yes | Yes |  |  |
| *Bathyergus suillus* | S7* | F |  | 3 | UP | H | U |  |  | Yes | Yes |  |  |
| *Bathyergus suillus* | S8 | F |  | 3 | UP | H | U |  |  | Yes | Yes |  |  |
| *Bathyergus suillus* | F1 | M |  | 3 | UP | H | U | F |  | Yes | Yes | Yes |  |
| *Bathyergus suillus* | F4 | M |  | 3 | UP | H | U | F |  | Yes | Yes | Yes |  |
| *Bathyergus suillus* | S1* | M |  | 3 | UP | H | U | F |  | Yes | Yes | Yes |  |
| *Bathyergus suillus* | S15* | M |  | 3 | UP | H | U | F |  | Yes | Yes | Yes |  |
| *Bathyergus suillus* | S18 | M |  | 3 | UP | H | U | F |  | Yes | Yes | Yes |  |
| *Bathyergus suillus* | S19 | M |  | 3 | UP | H | U | F |  | Yes | Yes | Yes |  |
| *Bathyergus suillus* | S4 | M |  | 3 | UP | H | U | F |  | Yes | Yes | Yes |  |
| *Bathyergus suillus* | S5 | M |  | 3 | UP | H | U | F |  | Yes | Yes | Yes |  |
| *Bathyergus suillus* | S9 | M |  | 3 | UP | H | U | F |  | Yes | Yes | Yes |  |
| *Bathyergus suillus* | GM282* | F |  | 3 | UP | H | U | F | T-Fi | Yes | Yes | Yes | Yes |
| *Bathyergus suillus* | GM283* | F |  | 3 | UP | H | U | F | T-Fi | Yes | Yes | Yes | Yes |
| *Bathyergus suillus* | GM284* | M |  | 3 | UP | H | U | F | T-Fi | Yes | Yes | Yes | Yes |
| *Bathyergus suillus* | GM285* | F |  | 3 | UP | H | U | F | T-Fi | Yes | Yes | Yes | Yes |
| *Bathyergus janetta* | GM005 | F |  | 3 | UCT | H | U | F | T-Fi | Yes | Yes | Yes | Yes |
| *Bathyergus janetta* | GM007 | M |  | 3 | UCT | H | U | F | T-Fi | Yes | Yes | Yes | Yes |
| *Bathyergus janetta* | GM013 | F |  | 3 | UCT | H |  | F |  | Yes |  | Yes |  |
| *Bathyergus janetta* | GM014 | M |  | 3 | UCT | H |  | F |  | Yes |  | Yes |  |
| *Bathyergus janetta* | GM239 | - | 2 days | 1 | UCT | H | U | F | T-Fi | Yes | Yes | Yes | Yes |
| *Bathyergus janetta* | GM503 | - |  | 2? | UCT | H | U | F | T-Fi | Yes | Yes | Yes | Yes |
| *Heliophobius argenteocinereus* | GM245 | M | a few days |  | UZ | H | U | F | T-Fi | Yes | Yes | Yes | No |
| *Heliophobius argenteocinereus* | GM246 | F | 1 day |  | UZ | H | U |  |  | Yes | Yes |  |  |
| *Heliophobius argenteocinereus* | GM247 | M | ~4 days |  | UZ | H | U | F | T-Fi | Yes | Yes | Yes | Yes |
| *Heliophobius argenteocinereus* | GM248 | F | ~14 days |  | UZ | H | U | F | T-Fi | Yes | Yes | Yes | Yes |
| *Heliophobius argenteocinereus* | 103 | M | 2 months |  | USB | H | U | F | T-Fi | Yes | Yes | Yes | No |
| *Heliophobius argenteocinereus* | 77 | M | 2 months |  | USB | H | U | F | T-Fi | Yes | Yes | Yes | Yes |
| *Heliophobius argenteocinereus* | 20 | M | 3.5 months |  | USB | H | U | F | T-Fi | Yes | Yes | Yes | Yes |
| *Heliophobius argenteocinereus* | 350* | F | 1 month |  | USB | H | U | F | T-Fi | Yes | Yes | No | Yes |
| *Heliophobius argenteocinereus* | 526 | M | 2 years, 4 months | 3 | USB | H | U | F | T-Fi | Yes | Yes | Yes | Yes |
| *Heliophobius argenteocinereus* | 26* | F | 1.5 months |  | USB | H | U | F | T-Fi | Yes | Yes | Yes | Yes |
| *Heliophobius argenteocinereus* | 11 | F | 2.5 months |  | USB | H | U | F | T-Fi | Yes | Yes | Yes | Yes |
| *Heliophobius argenteocinereus* | 387* | F | at least 5 years | 3 | USB | H | U | F | T-Fi | Yes | Yes | Yes | Yes |
| *Heliophobius argenteocinereus* | 27 | F | 2 years, 10 months | 3 | USB | H | U | F | T-Fi | Yes | Yes | Yes | Yes |
| *Heliophobius argenteocinereus* | 386 | M | at least 6 years | 3 | USB | H | U | F | T-Fi | Yes | Yes | Yes | Yes |
| *Heliophobius argenteocinereus* | 8 | M | 4 years, 9 months | 3 | USB | H | U | F | T-Fi | Yes | Yes | Yes | Yes |
| *Heliophobius argenteocinereus* | 6* | F | at least 1 year, 3 months |  | USB | H | U | F | T-Fi | Yes | Yes | Yes | Yes |
| *Heliophobius argenteocinereus* | 516 | M | at least 8 years, 2 months | 3 | USB | H | U | F | T-Fi | Yes | Yes | Yes | Yes |
| *Heliophobius argenteocinereus* | 361* | F | at least 2 years, 1 month | 3 | USB | H | U | F | T-Fi | Yes | Yes | Yes | Yes |
| *Heliophobius argenteocinereus* | 517 | M | at least 2 years, 7 months | 3 | USB | H | U | F | T-Fi | Yes | Yes | Yes | Yes |
| *Heliophobius argenteocinereus* | 585 | M | at least 5 years, 7 months | 3 | USB | H | U | F | T-Fi | Yes | Yes | Yes | Yes |
| *Heliophobius argenteocinereus* | 377 | M | at least 2 years, 2 months | 3 | USB | H | U | F | T-Fi | Yes | Yes | Yes | Yes |
| *Heliophobius argenteocinereus* | 451 | F | at least 2 years, 1 month | 3 | USB | H | U | F | T-Fi | Yes | Yes | Yes | Yes |
| *Heliophobius argenteocinereus* | 5* | F | at least 3 years | 3 | USB | H | U | F | T-Fi | Yes | Yes | Yes | Yes |
| *Heliophobius argenteocinereus* | 18 | M | at least 6 years, 8 months | 3 | USB | H | U | F | T-Fi | Yes | Yes | Yes | Yes |
| *Heliophobius argenteocinereus* | 393 | M | at least 1 year, 11 months |  | USB | H | U | F | T-Fi | Yes | Yes | Yes | Yes |
| *Heliophobius argenteocinereus* | 473 | F | at least 8 years | 3 | USB | H | U | F | T-Fi | Yes | Yes | Yes | Yes |
| *Heliophobius argenteocinereus* | 476 | M | at least 2 years, 10 months | 3 | USB | H | U | F | T-Fi | Yes | Yes | Yes | Yes |
| *Heliophobius argenteocinereus* | 508 | F | at least 3 years, 8 months | 3 | USB | H | U | F | T-Fi | Yes | Yes | Yes | Yes |
| *Heliophobius argenteocinereus* | 525 | F | 2 years, 5 months | 3 | USB | H | U | F | T-Fi | Yes | Yes | Yes | Yes |
| *Heliophobius argenteocinereus* | 492 | F | at least 14 years | 3 | USB | H | U | F | T-Fi | Yes | Yes | Yes | Yes |
| *Heliophobius argenteocinereus* | 299* | F | 2-3 days old |  | USB | H | U | F | T-Fi | Yes | Yes | Yes | No |
| *Heliophobius argenteocinereus* | 479* | F | 6 years, 10 months | 3 | USB | H | U | F | T-Fi | Yes | Yes | Yes | Yes |
| *Heliophobius argenteocinereus* | 244 | M | at least 2 years, 1 month | 3 | USB | H | U | F | T-Fi | Yes | Yes | Yes | Yes |
| *Heliophobius argenteocinereus* | 590* | F | at least 6 years | 3 | USB | H | U | F | T-Fi | Yes | Yes | Yes | Yes |
| *Heliophobius argenteocinereus* | NB10* | M |  | 3 | UP | H | U | F | T-Fi | Yes | Yes | Yes | Yes |
| *Heliophobius argenteocinereus* | NB21* | F |  | 3 | UP | H | U | F | T-Fi | Yes | Yes | Yes | Yes |
| *Heliophobius argenteocinereus* | NB05* | M |  | 3 | UP | H | U | F | T-Fi | Yes | Yes | Yes | Yes |
| *Heliophobius argenteocinereus* | NB15 | F |  | 3 | UP | H | U | F | T-Fi | Yes | Yes | Yes | Yes |
| *Georychus capensis* | GM528 |  | ~2 months | 2 | UCT | H | U | F | T-Fi | Yes | Yes | Yes | Yes |
| *Georychus capensis* | GM530 |  | 3 days | 1 | UCT | H | U |  |  | Yes | Yes |  |  |
| *Georychus capensis* | JO475* | F | NB21 | 2 | UP | H | U | F | T-Fi | Yes | Yes | Yes | Yes |
| *Georychus capensis* | JO402* | M | GM530 | 3 | UP | H | U | F | T-Fi | Yes | Yes | Yes | Yes |
| *Georychus capensis* | GM295* | - | JO402 | 2 | UCT | H | U | F | T-Fi | Yes | Yes | Yes | Yes |
| *Georychus capensis* | GM296* | F | GM295 | 1 | UP | H | U | F | T-Fi | Yes | Yes | Yes | Yes |
| *Georychus capensis* | GM297* | F | GM296 | 2 | UP | H | U | F | T-Fi | Yes | Yes | Yes | Yes |
| *Georychus capensis* | GM298* | F | GM297 | 2 | UP | H | U | F | T-Fi | Yes | Yes | Yes | Yes |
| *Georychus capensis* | Z4 | F | GM298 | 2 | UP | H | U |  |  | Yes | Yes |  |  |
| *Georychus capensis* | Z6 | F | Z4 | 3 | UP | H | U |  |  | Yes | Yes |  |  |
| *Georychus capensis* | Z9 | M | Z9 | 2 | UP | H | U |  |  | Yes | Yes |  |  |
| *Georychus capensis* | Z10 | F | Z10 | 2 | UP | H | U |  |  | Yes | Yes |  |  |
| *Georychus capensis* | Z11 | F | Z11 | 3 | UP | H | U |  |  | Yes | Yes |  |  |
| *Georychus capensis* | Z12 | F | Z12 | 3 | UP | H | U |  |  | Yes | Yes |  |  |
| *Georychus capensis* | Z22 | M | Z22 | 1 | UP | H | U |  |  | Yes | Yes |  |  |
| *Georychus capensis* | Z23 | F | Z23 | 2 | UP | H | U |  |  | Yes | Yes |  |  |
| *Georychus capensis* | Z25 | F | Z25 | 3 | UP | H | U |  |  | Yes | Yes |  |  |
| *Georychus capensis* | Z27 | F | Z27 | 1 | UP | H | U |  |  | Yes | Yes |  |  |
| *Georychus capensis* | Z28 | M | Z28 | 1 | UP | H | U |  |  | Yes | Yes |  |  |
| *Georychus capensis* | Z31 | F | Z31 | 3 | UP | H | U |  |  | Yes | Yes |  |  |
| *Georychus capensis* | Z32 | F | Z32 | 2 | UP | H | U |  |  | Yes | Yes |  |  |
| *Georychus capensis* | Z33 | M | Z33 | 2 | UP | H | U |  |  | Yes | Yes |  |  |
| *Georychus capensis* | Z35 | F | Z35 | 3 | UP | H | U |  |  | Yes | Yes |  |  |
| *Georychus capensis* | Z36 | F | Z36 | 2 | UP | H | U |  |  | Yes | Yes |  |  |
| *Georychus capensis* | Z37 | F | Z37 | 3 | UP | H | U |  |  | Yes | Yes |  |  |
| *Georychus capensis* | Z38 | F | Z38 | 3 | UP | H | U |  |  | Yes | Yes |  |  |
| *Georychus capensis* | Z210 | M | Z210 | 1 | UP | H | U |  |  | Yes | Yes |  |  |
| *Georychus capensis* | Z212 | M | Z212 | 2 | UP | H | U |  |  | Yes | Yes |  |  |
| *Georychus capensis* | Z214 | F | Z214 | 3 | UP | H | U |  |  | Yes | Yes |  |  |
| *Georychus capensis* | Z215 | M | Z215 | 2 | UP | H |  |  |  | Yes |  |  |  |
| *Georychus capensis* | Z216 | F | Z216 | 1 | UP | H | U |  |  | Yes | Yes |  |  |
| *Georychus capensis* | Z218 | F | Z225 | 2 | UP |  | U |  |  |  | Yes |  |  |
| *Georychus capensis* | Z219 | M | A01 | 2 | UP | H | U |  |  | Yes | Yes |  |  |
| *Georychus capensis* | Z225 | - | A03 | 2 | UP | H | U |  |  | Yes | Yes |  |  |
| *Georychus capensis* | A01 | F | A06 | 1 | UP | H | U | F | T-Fi | Yes | Yes | Yes | Yes |
| *Georychus capensis* | A03 | F | A19 | 1 | UP | H | U | F | T-Fi | Yes | Yes | Yes | Yes |
| *Georychus capensis* | A06 | M | A35 | 1 | UP | H | U | F | T-Fi | Yes | Yes | Yes | Yes |
| *Georychus capensis* | A19 | M | A40 | 2 | UP | H | U | F | T-Fi | Yes | Yes | Yes | Yes |
| *Georychus capensis* | A35 | F | A43 | 2 | UP | H | U | F | T-Fi | Yes | Yes | Yes | Yes |
| *Georychus capensis* | A40 | M | A51 | 2 | UP | H | U | F | T-Fi | Yes | Yes | Yes | Yes |
| *Georychus capensis* | A43 | F | A95 | 2 | UP | H | U | F | T-Fi | Yes | Yes | Yes | Yes |
| *Georychus capensis* | A51 | F | A97 | 2 | UP | H | U | F | T-Fi | Yes | Yes | Yes | Yes |
| *Georychus capensis* | A95 | F | A99 | 3 | UP | H | U | F | T-Fi | Yes | Yes | Yes | Yes |
| *Georychus capensis* | A97 | F | A100 | 3 | UP | H | U | F | T-Fi | Yes | Yes | Yes | Yes |
| *Georychus capensis* | A99 | F | A115 | 3 | UP | H | U | F | T-Fi | Yes | Yes | Yes | Yes |
| *Georychus capensis* | A100 | F | A118 | 3 | UP | H | U | F | T-Fi | Yes | Yes | Yes | Yes |
| *Georychus capensis* | A115 | M | A128 | 3 | UP | H | U | F | T-Fi | Yes | Yes | Yes | Yes |
| *Georychus capensis* | A118 | M | A130 | 3 | UP | H | U | F | T-Fi | Yes | Yes | Yes | Yes |
| *Georychus capensis* | A128 | F | A131 | 3 | UP | H | U | F | T-Fi | Yes | Yes | Yes | Yes |
| *Georychus capensis* | A130 | F |  | 3 | UP | H | U | F | T-Fi | Yes | Yes | Yes | Yes |
| *Georychus capensis* | A131 | F |  | 2 | UP | H | U | F | T-Fi | Yes | Yes | Yes | Yes |
| *Cryptomys hottentotus* | GM102 | F |  | 3 | UCT | H | U | F | T-Fi | Yes | Yes | Yes | Yes |
| *Cryptomys hottentotus* | GM103 | M |  | 3 | UCT | H | U | F | T-Fi | Yes | Yes | Yes | Yes |
| *Cryptomys hottentotus* | GM104 | M |  | 3 | UCT | H | U | F | T-Fi | Yes | Yes | Yes | Yes |
| *Cryptomys hottentotus* | GM105 | M |  | 3 | UCT | H | U | F | T-Fi | Yes | Yes | Yes | Yes |
| *Cryptomys hottentotus* | GM106* | M |  | 3 | UCT | H | U | F | T-Fi | Yes | Yes | Yes | Yes |
| *Cryptomys hottentotus* | GM107 | M |  | 2 | UCT | H | U | F | T-Fi | Yes | Yes | Yes | Yes |
| *Cryptomys hottentotus* | GM108 | F |  | 3 | UCT | H | U | F | T-Fi | Yes | Yes | Yes | Yes |
| *Cryptomys hottentotus* | GM109 | M |  | 3 | UCT | H | U | F | T-Fi | Yes | Yes | Yes | Yes |
| *Cryptomys hottentotus* | GM110 | M |  | 3 | UCT | H | U | F | T-Fi | Yes | Yes | Yes | Yes |
| *Cryptomys hottentotus* | GM111 | F |  | 3 | UCT | H | U | F | T-Fi | Yes | Yes | Yes | Yes |
| *Cryptomys hottentotus* | GM112 | F |  | 3 | UCT | H | U | F | T-Fi | Yes | Yes | Yes | Yes |
| *Cryptomys hottentotus* | GM113 | F |  | 2 | UCT | H | U | F | T-Fi | Yes | Yes | Yes | Yes |
| *Cryptomys hottentotus* | GM114 | F |  | 2 | UCT | H | U | F | T-Fi | Yes | Yes | Yes | Yes |
| *Cryptomys hottentotus* | GM115* | M |  | 3 | UCT | H | U | F | T-Fi | Yes | Yes | Yes | Yes |
| *Cryptomys hottentotus* | GM116 | - |  | 3 | UCT | H | U | F | T-Fi | Yes | Yes | Yes | Yes |
| *Cryptomys hottentotus* | GM117 | M |  | 3 | UCT | H | U | F | T-Fi | Yes | Yes | Yes | Yes |
| *Cryptomys hottentotus* | GM118 | F |  | 2 | UCT | H | U | F | T-Fi | Yes | Yes | Yes | Yes |
| *Cryptomys hottentotus* | GM119* | M |  | 2 | UCT | H | U | F | T-Fi | Yes | Yes | Yes | Yes |
| *Cryptomys hottentotus* | GM120 | F |  | 2 | UCT | H | U | F | T-Fi | Yes | Yes | Yes | Yes |
| *Cryptomys hottentotus* | GM121* | M |  | 2 | UCT | H | U | F | T-Fi | Yes | Yes | Yes | Yes |
| *Cryptomys hottentotus* | GM122 | F |  | 3 | UCT | H | U | F | T-Fi | Yes | Yes | Yes | Yes |
| *Cryptomys hottentotus* | GM123* | M |  | 3 | UCT | H | U | F | T-Fi | Yes | Yes | Yes | Yes |
| *Cryptomys hottentotus* | GM124 | F |  | 2 | UCT | H | U | F | T-Fi | Yes | Yes | Yes | Yes |
| *Cryptomys hottentotus* | GM125 | F |  | 3 | UCT | H | U | F | T-Fi | Yes | Yes | Yes | Yes |
| *Cryptomys hottentotus* | GM126 | M |  | 3 | UCT | H | U | F | T-Fi | Yes | Yes | Yes | Yes |
| *Cryptomys hottentotus* | GM127 | M |  | 3 | UCT | H | U | F | T-Fi | Yes | Yes | Yes | Yes |
| *Cryptomys hottentotus* | GM128 | F |  | 3 | UCT | H | U | F | T-Fi | Yes | Yes | Yes | Yes |
| *Cryptomys hottentotus* | GM129* | M |  | 3 | UCT | H | U | F | T-Fi | Yes | Yes | Yes | Yes |
| *Cryptomys hottentotus* | GM130 | F |  | 2 | UCT | H | U | F | T-Fi | Yes | Yes | Yes | Yes |
| *Cryptomys hottentotus* | GM131 | M |  | 2 | UCT | H | U | F | T-Fi | Yes | Yes | Yes | Yes |
| *Cryptomys hottentotus* | GM132* | M |  | 3 | UCT | H | U | F | T-Fi | Yes | Yes | Yes | Yes |
| *Cryptomys hottentotus* | GM133 | F |  | 3 | UCT | H | U | F | T-Fi | Yes | Yes | Yes | Yes |
| *Cryptomys hottentotus* | GM134 | M |  | 2 | UCT | H | U | F | T-Fi | Yes | Yes | Yes | Yes |
| *Cryptomys hottentotus* | GM135 | M |  | 3 | UCT | H | U | F | T-Fi | Yes | Yes | Yes | Yes |
| *Cryptomys hottentotus* | GM136 | F |  | 2 | UCT | H | U | F | T-Fi | Yes | Yes | Yes | Yes |
| *Cryptomys hottentotus* | GM137 | M |  | 2 | UCT | H | U | F | T-Fi | Yes | Yes | Yes | Yes |
| *Cryptomys hottentotus* | GM138 | M |  | 3 | UCT | H | U | F | T-Fi | Yes | Yes | Yes | Yes |
| *Cryptomys hottentotus* | GM139 | M |  | 2 | UCT | H | U | F | T-Fi | Yes | Yes | Yes | Yes |
| *Cryptomys hottentotus* | GM140 | M |  | 3 | UCT | H | U | F | T-Fi | Yes | Yes | Yes | Yes |
| *Cryptomys hottentotus* | GM141 | F |  | 3 | UCT | H | U | F | T-Fi | Yes | Yes | Yes | Yes |
| *Cryptomys hottentotus* | GM142 | M |  | 3 | UCT | H | U | F | T-Fi | Yes | Yes | Yes | Yes |
| *Cryptomys hottentotus* | GM143 | F |  | 3 | UCT | H | U | F | T-Fi | Yes | Yes | Yes | Yes |
| *Cryptomys hottentotus* | GM144 | F |  | 3 | UCT | H | U | F | T-Fi | Yes | Yes | Yes | Yes |
| *Cryptomys hottentotus* | GM145* | M |  | 3 | UCT | H | U | F | T-Fi | Yes | Yes | Yes | Yes |
| *Cryptomys hottentotus* | GM146 | F |  | 3 | UCT | H | U | F | T-Fi | Yes | Yes | Yes | Yes |
| *Cryptomys hottentotus* | GM147 | F |  | 3 | UCT | H | U | F | T-Fi | Yes | Yes | Yes | Yes |
| *Cryptomys hottentotus* | GM148 | M |  | 2 | UCT | H | U | F | T-Fi | Yes | Yes | Yes | Yes |
| *Cryptomys hottentotus* | GM149 | F |  | 2 | UCT | H | U | F | T-Fi | Yes | Yes | Yes | Yes |
| *Cryptomys hottentotus* | GM150* | M |  | 3 | UCT | H | U | F | T-Fi | Yes | Yes | Yes | Yes |
| *Cryptomys hottentotus* | GM151 | F |  | 3 | UCT | H | U | F | T-Fi | Yes | Yes | Yes | Yes |
| *Cryptomys hottentotus* | GM152* | M |  | 3 | UCT | H | U | F | T-Fi | Yes | Yes | Yes | Yes |
| *Cryptomys hottentotus* | GM153 | - |  | 2 | UCT | H | U | F | T-Fi | Yes | Yes | Yes | Yes |
| *Cryptomys hottentotus* | GM154 | F |  | 2 | UCT | H | U | F | T-Fi | Yes | Yes | Yes | Yes |
| *Fukomys mechowii* | 227 | F | 1 day | 1 | USB | H | U | F | T-Fi | Yes | Yes | Yes | No |
| *Fukomys mechowii* | 228* | M | 1 day | 1 | USB | H | U | F | T-Fi | Yes | Yes | Yes | No |
| *Fukomys mechowii* | 146 | F | 2 days | 1 | USB | H | U | F | T-Fi | Yes | Yes | Yes | No |
| *Fukomys mechowii* | 148 | M | 1 day | 1 | USB | H | U | F | T-Fi | Yes | Yes | Yes | No |
| *Fukomys mechowii* | 145 | F | 5 days | 1 | USB | H | U | F | T-Fi | Yes | Yes | Yes | Yes |
| *Fukomys mechowii* | 147 | F | 3 days | 1 | USB | H | U | F | T-Fi | Yes | Yes | Yes | Yes |
| *Fukomys mechowii* | 59 | M | 2 days | 1 | USB | H | U | F | T-Fi | Yes | Yes | Yes | No |
| *Fukomys mechowii* | 154 | F | 2 days | 1 | USB | H | U | F | T-Fi | Yes | Yes | Yes | Yes |
| *Fukomys mechowii* | 55 | F | 4 days | 1 | USB | H | U | F | T-Fi | Yes | Yes | Yes | No |
| *Fukomys mechowii* | 58 | M | 4 days | 1 | USB | H | U | F | T-Fi | Yes | Yes | Yes | Yes |
| *Fukomys mechowii* | 125 | F | 1 month | 1 | USB | H | U | F | T-Fi | Yes | Yes | Yes | Yes |
| *Fukomys mechowii* | 167 | M | 21 days | 1 | USB | H | U | F | T-Fi | Yes | Yes | Yes | Yes |
| *Fukomys mechowii* | 36* | M | 5 days | 1 | USB | H | U | F | T-Fi | Yes | Yes | Yes | Yes |
| *Fukomys mechowii* | 45 | F | 9 days | 1 | USB | H | U | F | T-Fi | Yes | Yes | Yes | Yes |
| *Fukomys mechowii* | 153* | M | 21 days | 1 | USB | H | U | F | T-Fi | Yes | Yes | Yes | Yes |
| *Fukomys mechowii* | 78* | M | 1 month, 11 days | 1 | USB | H | U | F | T-Fi | Yes | Yes | Yes | Yes |
| *Fukomys mechowii* | 123 | F | 4-5 months? | 2 | USB | H | U | F | T-Fi | Yes | Yes | Yes | Yes |
| *Fukomys mechowii* | 54 | F | 17 days | 1 | USB | H | U | F | T-Fi | Yes | Yes | Yes | Yes |
| *Fukomys mechowii* | 116 | F |  | 1 | USB | H | U | F | T-Fi | Yes | Yes | Yes | No |
| *Fukomys mechowii* | 14* | M | 4 months | 2 | USB | H | U | F | T-Fi | Yes | Yes | Yes | Yes |
| *Fukomys mechowii* | 23 | F | 15 months | 2 | USB | H | U | F | T-Fi | Yes | Yes | Yes | Yes |
| *Fukomys mechowii* | 179* | M | 13 months | 2 | USB | H | U | F | T-Fi | Yes | Yes | Yes | Yes |
| *Fukomys mechowii* | 3 | F | 1 year, 10 months | 3 | USB | H | U | F | T-Fi | Yes | Yes | Yes | Yes |
| *Fukomys mechowii* | 1 | F | 4 years, 4 months | 3 | USB | H | U | F | T-Fi | Yes | Yes | Yes | Yes |
| *Fukomys mechowii* | 177* | M | 2 years, 3 months | 3 | USB | H | U | F | T-Fi | Yes | Yes | Yes | Yes |
| *Fukomys mechowii* | 497 | F | 13 years, 6 months | 3 | USB | H | U | F | T-Fi | Yes | Yes | Yes | Yes |
| *Fukomys mechowii* | 478* | M | 3 years, 2 months | 3 | USB | H | U | F | T-Fi | Yes | Yes | Yes | Yes |
| *Fukomys mechowii* | 494 | F | 7 years, 1 month | 3 | USB | H | U | F | T-Fi | Yes | Yes | Yes | Yes |
| *Fukomys mechowii* | 589 | F | 10 years, 7 months | 3 | USB | H | U | F | T-Fi | Yes | Yes | Yes | Yes |
| *Fukomys mechowii* | 242* | M | 3 years | 3 | USB | H | U | F | T-Fi | Yes | Yes | Yes | Yes |
| *Fukomys mechowii* | 2* | M | 1 year, 8 months | 3 | USB | H | U | F | T-Fi | Yes | Yes | Yes | Yes |
| *Fukomys mechowii* | 531 | - | 1 year, 5 months | 3 | USB | H | U | F | T-Fi | Yes | Yes | Yes | Yes |
| *Fukomys damarensis* | NB227 | F |  | 3 | UP | H | U | F | T-Fi | Yes | Yes | Yes | Yes |
| *Fukomys damarensis* | NB228 | F |  | 3 | UP | H | U | F | T-Fi | Yes | Yes | Yes | Yes |
| *Fukomys damarensis* | NB229 | F |  | 2 | UP | H | U | F | T-Fi | Yes | Yes | Yes | Yes |
| *Fukomys damarensis* | NB230* | M |  | 1-2? | UP | H | U | F | T-Fi | Yes | Yes | Yes | Yes |
| *Fukomys damarensis* | NB231* | M |  | 2 | UP | H | U | F | T-Fi | Yes | Yes | Yes | Yes |
| *Fukomys damarensis* | NB232* | M |  | 1 | UP | H | U | F | T-Fi | Yes | Yes | Yes | Yes |
| *Fukomys damarensis* | NB233* | M |  | 1 | UP | H | U | F | T-Fi | Yes | Yes | Yes | Yes |
| *Fukomys damarensis* | NB238 | - |  | 3 | UP | H | U |  |  | Yes | Yes |  |  |
| *Fukomys damarensis* | NB422 | M | 1 day | 1 | UP | H | U | F | T-Fi | Yes | Yes | Yes | Yes |
| *Fukomys damarensis* | NB423 | - | 1 day | 1 | UP | H | U | F | T-Fi | Yes | Yes | Yes | No |
| *Fukomys damarensis* | GAP315 | F |  | 3 | UCT | H | U |  |  | Yes | Yes |  |  |
| *Fukomys damarensis* | D11 | M |  | 3 | UP | H | U |  |  | Yes | Yes |  |  |
| *Fukomys damarensis* | D12 | M |  | 3 | UP | H | U |  |  | Yes | Yes |  |  |
| *Fukomys damarensis* | D13 | F |  | 3 | UP | H | U |  |  | Yes | Yes |  |  |
| *Fukomys damarensis* | D14 | M |  | 3 | UP | H | U |  |  | Yes | Yes |  |  |
| *Fukomys damarensis* | D15 | F |  | 3 | UP | H | U |  |  | Yes | Yes |  |  |
| *Fukomys damarensis* | D16 | M |  | 3 | UP | H | U |  |  | Yes | Yes |  |  |
| *Fukomys damarensis* | D17 | M |  | 3 | UP | H | U |  |  | Yes | Yes |  |  |
| *Fukomys damarensis* | D18 | F |  | 3 | UP | H | U |  |  | Yes | Yes |  |  |
| *Fukomys damarensis* | D19 | F |  | 3 | UP | H | U |  |  | Yes | Yes |  |  |
| *Fukomys damarensis* | HD2 | - |  | 2-3? | UP | H | U |  |  | Yes | Yes |  |  |
| *Fukomys damarensis* | FD5 | - |  | 2 | UP | H | U |  |  | Yes | Yes |  |  |
| *Fukomys damarensis* | GM039 | - |  | 1 | UP | H |  |  |  | Yes |  |  |  |
| *Fukomys damarensis* (ca) | G3F035 | F | 1 Day | 1 | KRC | H | U | F | T-Fi | Yes | Yes | Yes | No |
| *Fukomys damarensis* (ca) | G3M036* | M | 1 Day | 1 | KRC | H | U | F | T-Fi | Yes | Yes | Yes | Yes |
| *Fukomys damarensis* (ca) | G4F030 | F | 1 Month, 29 Days | 1 | KRC | H | U | F | T-Fi | Yes | Yes | Yes | Yes |
| *Fukomys damarensis* (ca) | G4F042 | F | 13 Months, 10 Days | 3 | KRC | H | U | F | T-Fi | Yes | Yes | Yes | Yes |
| *Fukomys damarensis* (ca) | G4M018 | M | 30 Months, 28 Days | 3 | KRC | H | U | F | T-Fi | Yes | Yes | Yes | Yes |
| *Fukomys damarensis* (ca) | G4M029 | M | 1 Month, 10 Days | 1 | KRC | H | U | F | T-Fi | Yes | Yes | Yes | Yes |
| *Fukomys damarensis* (ca) | G4M034* | M | 20 Months, 27 Days | 3 | KRC | H | U | F | T-Fi | Yes | Yes | Yes | Yes |
| *Fukomys damarensis* (ca) | G4M036 | M | 1 Month, 29 Days | 1 | KRC | H | U | F | T-Fi | Yes | Yes | Yes | Yes |
| *Fukomys damarensis* (ca) | G4M039* | M | 18 Months, 21 Days | 3 | KRC | H | U | F | T-Fi | Yes | Yes | Yes | Yes |
| *Fukomys damarensis* (ca) | G4M041* | M | 10 Months, 14 Days | 2 | KRC | H | U | F | T-Fi | Yes | Yes | Yes | Yes |
| *Fukomys damarensis* (ca) | G4M047* | M | 7 Months, 0 Days | 2 | KRC | H | U | F | T-Fi | Yes | Yes | Yes | Yes |
| *Fukomys damarensis* | F2M012* | M |  | 1 | KRC | H | U | F | T-Fi | Yes | Yes | Yes | Yes |
| *Fukomys damarensis* | F2F013 | F |  | 1 | KRC | H | U | F | T-Fi | Yes | Yes | Yes | Yes |
| *Fukomys damarensis* | G7F013 | F |  | 2 | KRC | H | U | F | T-Fi | Yes | Yes | Yes | Yes |
| *Fukomys damarensis* | F6F002 | F |  | 3 | KRC | H | U | F | T-Fi | Yes | Yes | Yes | Yes |
| *Fukomys damarensis* | G4M008* | M |  | 3 | KRC | H | U | F | T-Fi | Yes | Yes | Yes | Yes |
| *Fukomys damarensis* | G5F003 | F |  | 3 | KRC | H | U | F | T-Fi | Yes | Yes | Yes | Yes |
| *Fukomys damarensis* | L29F002 | F |  | 3 | KRC | H | U | F | T-Fi | Yes | Yes | Yes | Yes |
| *Fukomys damarensis* | G4F012 | F |  | 3 | KRC | H | U | F | T-Fi | Yes | Yes | Yes | Yes |
| *Fukomys damarensis* | G4F002 | F |  | 3 | KRC | H | U | F | T-Fi | Yes | Yes | Yes | Yes |
| *Fukomys damarensis* | G4M009* | M |  | 3 | KRC | H | U | F | T-Fi | Yes | Yes | Yes | Yes |
| *Fukomys damarensis* | G3M007 | M |  | 3 | KRC | H | U | F | T-Fi | Yes | Yes | Yes | Yes |
| *Fukomys damarensis* | G4M001 | M |  | 3 | KRC | H | U | F | T-Fi | Yes | Yes | Yes | Yes |
| *Fukomys damarensis* | G3M001* | M |  | 3 | KRC | H | U | F | T-Fi | Yes | Yes | Yes | Yes |
| *Fukomys damarensis* | Z3M006* | M |  | 3 | KRC | H | U | F | T-Fi | Yes | Yes | Yes | Yes |
| *Heterocephalus glaber* (ca) | JJ-402 | - | 3 days | 1 | UCT | H | U | F | T-Fi | No | Yes | Yes | No |
| *Heterocephalus glaber* (ca) | JJ-405 | - | Perinatal | 0 | UCT | H | U | F | T-Fi | No | Yes | Yes | No |
| *Heterocephalus glaber* (ca) | JJ-416 | - | 2 Months, 2 days | 1-2? | UCT | H | U | F | T-Fi | No | Yes | Yes | No |
| *Heterocephalus glaber* (ca) | JJ-417 | - | 5 days | 1 | UCT | H | U | F | T-Fi | No | Yes | Yes | No |
| *Heterocephalus glaber* (ca) | JJ-557 | - | Juvenile | 1? | UCT | H | U | F | T-Fi | No | Yes | Yes | No |
| *Heterocephalus glaber* (ca) | JJ-559 | - | Perinatal | 0 | UCT | H | U | F | T-Fi | No | Yes | Yes | No |
| *Heterocephalus glaber* (ca) | JJ-560 | - | Perinatal | 0 | UCT | H | U | F | T-Fi | No | Yes | Yes | No |
| *Heterocephalus glaber* (ca) | JJ-001 | F | ~1 year, 6 months | 2 | UCT | H | U | F | T-Fi | No | Yes | Yes | No |
| *Heterocephalus glaber* (ca) | JJ-002 | - |  | 2 | UCT | H | U | F | T-Fi | No | Yes | Yes | No |
| *Heterocephalus glaber* (ca) | JJ-008 | F | ~10 years | 3 | UCT | H | U | F | T-Fi | No | Yes | Yes | No |
| *Heterocephalus glaber* (ca) | JJ-009 | M |  | 3 | UCT | H | U | F | T-Fi | No | Yes | Yes | No |
| *Heterocephalus glaber* (ca) | JJ-010 | - | ~4 months | 1 | UCT | H | U | F | T-Fi | No | Yes | Yes | No |
| *Heterocephalus glaber* (ca) | JJ-045* | M |  | 2 | UCT | H | U | F | T-Fi | No | Yes | Yes | No |
| *Heterocephalus glaber* (ca) | JJ-046* | F |  | 2 | UCT | H | U | F | T-Fi | No | Yes | Yes | No |
| *Heterocephalus glaber* (ca) | 1200-047 | - |  | 2 | UCT | H | U | F | T-Fi | No | Yes | Yes | No |
| *Heterocephalus glaber* (ca) | 1200-048 | M |  | 3 | UCT | H | U | F | T-Fi | No | Yes | Yes | No |
| *Heterocephalus glaber* (ca) | 1200-049 | F |  | 3 | UCT | H | U | F | T-Fi | No | Yes | Yes | No |
| *Heterocephalus glaber* (ca) | 1200-050 | M |  | 3 | UCT | H | U | F | T-Fi | No | Yes | Yes | No |
| *Heterocephalus glaber* (ca) | 1200-051 | F |  | 3 | UCT | H | U | F | T-Fi | No | Yes | Yes | No |
| *Heterocephalus glaber* (ca) | 1200-052* | M |  | 3 | UCT | H | U | F | T-Fi | No | Yes | Yes | No |
| *Heterocephalus glaber* (ca) | 1200-053 | M |  | 3 | UCT | H | U | F | T-Fi | No | Yes | Yes | No |
| *Heterocephalus glaber* (ca) | 1200-054 | F |  | 3 | UCT | H | U | F | T-Fi | No | Yes | Yes | No |
| *Heterocephalus glaber* (ca) | 1200-055 | M |  | 3 | UCT | H | U | F | T-Fi | No | Yes | Yes | No |
| *Heterocephalus glaber* (ca) | 1200-056 | - |  | 3 | UCT | H | U | F | T-Fi | No | Yes | Yes | No |
| *Heterocephalus glaber* (ca) | 1200-057 | M |  | 3 | UCT | H | U | F | T-Fi | No | Yes | Yes | No |
| *Heterocephalus glaber* (ca) | 1200-058 | M |  | 3 | UCT | H | U | F | T-Fi | No | Yes | Yes | No |
| *Heterocephalus glaber* (ca) | 1200-059 | F |  | 3 | UCT | H | U | F | T-Fi | No | Yes | Yes | No |
| *Heterocephalus glaber* (ca) | 1200-060* | M |  | 3 | UCT | H | U | F | T-Fi | No | Yes | Yes | No |
| *Heterocephalus glaber* (ca) | 1200-061* | M |  | 3 | UCT | H | U | F | T-Fi | No | Yes | Yes | No |
| *Heterocephalus glaber* (ca) | 1200-063 | F |  | 3 | UCT | H | U | F | T-Fi | No | Yes | Yes | No |
| *Heterocephalus glaber* (ca) | 1200-064 | F |  | 3 | UCT | H | U | F | T-Fi | No | Yes | Yes | No |
| *Heterocephalus glaber* (ca) | 1200-065 | F |  | 3 | UCT | H | U | F | T-Fi | No | Yes | Yes | No |
| *Heterocephalus glaber* (ca) | 1200-066 | - |  | 3 | UCT | H | U | F | T-Fi | No | Yes | Yes | No |
| *Heterocephalus glaber* (ca) | 1200-067* | F |  | 2 | UCT | H | U | F | T-Fi | No | Yes | Yes | No |
| *Heterocephalus glaber* (ca) | 1200-068 | F |  | 2 | UCT | H |  | F | T-Fi | No |  | Yes | No |
| *Heterocephalus glaber* (ca) | 1200-069 | - |  | 3 | UCT | H | U | F | T-Fi | No | Yes | Yes | Yes |
| *Heterocephalus glaber* (ca) | 1000-070 | F |  | 2 | UCT | H | U | F | T-Fi | No | Yes | Yes | No |
| *Heterocephalus glaber* (ca) | 1000-071 | F |  | 3 | UCT | H | U | F | T-Fi | No | Yes | Yes | No |
| *Heterocephalus glaber* (ca) | 1000-073 | M |  | 3 | UCT | H | U | F | T-Fi | No | Yes | Yes | No |
| *Heterocephalus glaber* (ca) | 1000-074 | M |  | 3 | UCT | H | U | F | T-Fi | No | Yes | Yes | No |
| *Heterocephalus glaber* (ca) | 1000-075 | - |  | 3 | UCT | H | U | F | T-Fi | No | Yes | Yes | No |
| *Heterocephalus glaber* (ca) | 1000-076 | F |  | 3 | UCT | H | U | F | T-Fi | No | Yes | Yes | No |
| *Heterocephalus glaber* (ca) | 1000-077 | F |  | 3 | UCT | H | U | F | T-Fi | No | Yes | Yes | No |
| *Heterocephalus glaber* (ca) | 1000-078 | F |  | 3 | UCT | H | U | F | T-Fi | No | Yes | Yes | No |
| *Heterocephalus glaber* (ca) | 1000-079 | F |  | 3 | UCT | H | U | F | T-Fi | No | Yes | Yes | Yes |
| *Heterocephalus glaber* (ca) | 1000-080 | F |  | 3 | UCT | H | U | F | T-Fi | No | Yes | Yes | No |
| *Heterocephalus glaber* (ca) | 1000-081* | M |  | 3 | UCT | H | U | F | T-Fi | No | Yes | Yes | Yes |
| *Heterocephalus glaber* (ca) | 1000-082* | M |  | 3 | UCT | H | U | F | T-Fi | No | Yes | Yes | No |
| *Heterocephalus glaber* (ca) | 1000-083 | F |  | 3 | UCT | H | U | F | T-Fi | No | Yes | Yes | No |
| *Heterocephalus glaber* (ca) | 1000-084 | F |  | 3 | UCT | H | U | F | T-Fi | No | Yes | Yes | No |
| *Heterocephalus glaber* (ca) | 1000-085 | M |  | 3 | UCT | H | U | F | T-Fi | No | Yes | Yes | No |
| *Heterocephalus glaber* (ca) | 1000-086 | F |  | 3 | UCT | H | U | F | T-Fi | No | Yes | Yes | No |
| *Heterocephalus glaber* (ca) | 5000-087 | M |  | 3 | UCT | H | U | F | T-Fi | No | Yes | Yes | No |
| *Heterocephalus glaber* (ca) | 5000-088* | M |  | 2 | UCT | H | U | F | T-Fi | No | Yes | Yes | No |
| *Heterocephalus glaber* (ca) | 5000-091 | F |  | 3 | UCT | H | U | F | T-Fi | No | Yes | Yes | No |
| *Heterocephalus glaber* (ca) | 5000-092* | M |  | 3 | UCT | H | U | F | T-Fi | No | Yes | Yes | No |
| *Heterocephalus glaber* (ca) | 5000-093 | F |  | 3 | UCT | H | U | F | T-Fi | No | Yes | Yes | No |
| *Heterocephalus glaber* (ca) | 5000-094 | M |  | 3 | UCT | H | U | F | T-Fi | No | Yes | Yes | No |
| *Heterocephalus glaber* (ca) | 5000-095 | M |  | 2 | UCT | H | U | F | T-Fi | No | Yes | Yes | No |
| *Heterocephalus glaber* (ca) | 5000-096 | F |  | 3 | UCT | H | U | F | T-Fi | No | Yes | Yes | No |
| *Heterocephalus glaber* (ca) | 5000-097 | F |  | 3 | UCT | H | U | F | T-Fi | No | Yes | Yes | No |
| *Heterocephalus glaber* (ca) | 5000-098 | F |  | 3 | UCT | H | U | F | T-Fi | No | Yes | Yes | No |
| *Heterocephalus glaber* (ca) | 5000-099 | M |  | 3 | UCT | H | U | F | T-Fi | No | Yes | Yes | No |
| *Heterocephalus glaber* (ca) | 5000-100 | F |  | 3 | UCT | H | U | F | T-Fi | No | Yes | Yes | No |
| *Heterocephalus glaber* (ca) | 5000-101* | M |  | 3 | UCT | H | U | F | T-Fi | No | Yes | Yes | No |
| *Heterocephalus glaber* (ca) | GM498 | F |  | 3 | UP | H | U | F | T-Fi | No | Yes | Yes | No |
| *Heterocephalus glaber* (ca) | GM499 | M |  | 3? | UP | H | U | F | T-Fi | No | Yes | Yes | No |
| *Heterocephalus glaber* (ca) | GM500 | ? |  | 3? | UP | H | U | F | T-Fi | No | Yes | Yes | No |
| *Heterocephalus glaber* (ca) | GM501 | F |  | 3? | UP | H | U | F | T-Fi | No | Yes | Yes | No |
| *Heterocephalus glaber* (ca) | GM505 | M |  | 3? | UP | H | U | F | T-Fi | No | Yes | Yes | No |
| *Heterocephalus glaber* (ca) | GM506 | F |  | 3 | UP | H | U | F | T-Fi | No | Yes | Yes | No |
| *Heterocephalus glaber* (ca) | GM507 | F |  | 3? | UP | H | U | F | T-Fi | No | Yes | Yes | No |
| *Heterocephalus glaber* (ca) | GM508 | F |  | 3? | UP | H | U | F | T-Fi | No | Yes | Yes | No |
| *Heterocephalus glaber* (ca) | GM509 | F |  | 3? | UP | H | U | F | T-Fi | No | Yes | Yes | No |
| *Heterocephalus glaber* (ca) | GM510 | F |  | 3? | UP | H | U | F | T-Fi | No | Yes | Yes | No |
| *Heterocephalus glaber* (ca) | GM511 | F |  | 3? | UP | H | U | F | T-Fi | No | Yes | Yes | No |
| *Hystrix africaeaustralis* | 38634 |  |  |  | IZIKO | H | U | F | T-Fi | Yes | Yes | No | No |
| *Hystrix africaeaustralis* | 36199 |  |  |  | IZIKO | H | U | F | T-Fi | Yes | Yes | No | No |
| *Hystrix africaeaustralis* | 38502 |  |  |  | IZIKO | H | U | F | T-Fi | Yes | Yes | No | No |
| *Hystrix africaeaustralis* | 38536 |  |  |  | IZIKO | H | U | F | T-Fi | Yes | Yes | No | No |
| *Hystrix africaeaustralis* | 36738 |  |  |  | IZIKO | H | U | F | T-Fi | Yes | Yes | No | No |
| *Hystrix africaeaustralis* | 35988 |  |  |  | IZIKO | H | U | F | T-Fi | Yes | Yes | No | No |
| *Hystrix africaeaustralis* | 39666 |  |  |  | IZIKO | H | U | F | T-Fi | Yes | Yes | No | No |
| *Hystrix africaeaustralis* | 40427 |  |  |  | IZIKO | H | U | F | T-Fi | Yes | Yes | No | No |
| *Hystrix africaeaustralis* | 37705 |  |  |  | IZIKO | H | U | F | T-Fi | Yes | Yes | No | No |
| *Hystrix africaeaustralis* | 40403 |  |  |  | IZIKO | H | U | F | T-Fi | Yes | Yes | No | No |
| *Hystrix africaeaustralis* | 35137 |  |  |  | IZIKO | H | U | F |  | Yes | Yes | No |  |
| *Hystrix africaeaustralis* | 40310 |  |  |  | IZIKO | H | U | F | T-Fi | Yes | Yes | No | No |
| *Hystrix africaeaustralis* | 36139 |  |  |  | IZIKO | H | U |  |  | Yes | Yes |  |  |
| *Hystrix africaeaustralis* | 38525 |  |  |  | IZIKO | H | U | F | T-Fi | Yes | Yes | No | No |
| *Hystrix africaeaustralis* | 40429 |  |  |  | IZIKO | H | U | F | T-Fi | Yes | Yes | No | No |
| *Hystrix africaeaustralis* | 40744 |  |  |  | IZIKO | H | U | F | T-Fi | Yes | Yes | No | No |
| *Hystrix africaeaustralis* | 36060 |  |  |  | IZIKO | H | U | F | T-Fi | Yes | Yes | No | No |
| *Hystrix africaeaustralis* | 38256 |  |  |  | IZIKO | H | U | F | T-Fi | Yes | Yes | No | No |
| *Petromus typicus* | 40790 |  |  |  | IZIKO | H | U | F | T-Fi | Yes | Yes | No | No |

**Supplementary Table 2.** Comparison of the out-of-sample predictive score by means of the Widely Applicable Information Criterion (WAIC) of models with and without body mass (BM). SE gives an approximate standard error of each score, *Δ_i_* WAIC gives the difference between the *i*th model and the model with the lowest WAIC, *Δ_i_* SE gives the approximate standard error of the difference between the *i*th model and the model with the lowest WAIC, and *wi* is the weight of the *i*th model and indicates a relative support for that model. Model with a subscript *I* indicates a model without BM (i.e. intercept-only), and model with a subscript BM is a model with BM. Relative position of the deltoid tuberosity (RDT); Index of fossorial ability (IFA); Tibio-fibular junction index (TJI).

| Index | Model | *WAIC* (*SE*) | *Δ_i_* *WAIC* | *Δ_i_* *SE* | *w_i_* |
| --- | --- | --- | --- | --- | --- |
| RDT |  |  |  |  |  |
|  | RDT*_I_* | -572.71 (16.62) | 0 | *NA* | 0.61 |
|  | RDT*_BM_* | -571.78 (16.86) | 0.93 | 2.38 | 0.39 |
| TJI |  |  |  |  |  |
|  | TJI*_I_* | -467.85 (20.04) | 0 | *NA* | 0.62 |
|  | TJI*_BM_* | -466.85 (21.03) | 1.01 | 3.02 | 0.38 |
| IFA |  |  |  |  |  |
|  | IFA*_BM_* | -627.95 (24.41) | 0 | *NA* | 0.96 |
|  | IFA*_I_* | -621.64 (24.64) | 6.31 | 4.43 | 0.04 |

**Supplementary Table 3**. Summary of the phylogenetic varying effects regression with estimates of population-level parameters for three morpho-functional indices. Model for each index includes estimate of intercept mean (*µ_α_*) and slope mean (*µ_β_*), as well as estimates of phylogenetic (*σ_phy_*) and species-specific (*σ_spp_*) components, with 89% compatibility intervals (CI) in square brackets. Relative position of the deltoid tuberosity (RDT); Index of fossorial ability (IFA); Tibio-fibular junction index (TJI).

| Index | *µ_α_* [89% CI] | *µ_β_* [89% CI] | $\sigma_{phy_{\alpha}}$ [89% CI] | $\sigma_{phy_{\beta}}$[89% CI] | $\sigma_{spp}$ [89% CI] |
| --- | --- | --- | --- | --- | --- |
| RDT | -0.58 [-0.70, -0.45] | 0.01 [-0.01, 0.03] | 0.02 [0.00, 0.09] | 0.00 [0.00, 0.00] | 0.07 [0.00, 0.19] |
| TJI | -0.68 [-0.82, -0.51] | 0.01 [-0.01, 0.04] | 0.03 [0.00, 0.13] | 0.00 [0.00, 0.00] | 0.08 [0.01, 0.24] |
| IFA | -1.31 [-1.49, -1.05] | 0.02 [-0.01, 0.06] | 0.06 [0.00, 0.23] | 0.00 [0.00, 0.01] | 0.11 [0.01, 0.33] |

**Supplementary Table 4.** Summary of the phylogenetic varying effects regression for three morpho-functional indices. Each species *j* has its own estimate of intercept (*α_j_*) and slope (*β_j_*), with 89% compatibility intervals (CI) in square brackets. Relative position of the deltoid tuberosity (RDT); Index of fossorial ability (IFA); Tibio-fibular junction index (TJI).

| Index | Species | *α_j_* [89% CI] | *β_j_* [89% CI] |
| --- | --- | --- | --- |
| RDT |  |  |  |
|  | *B. suillus* | -0.58 [-0.71, -0.45] | 0.01 [-0.01, 0.03] |
|  | *C. hottentotus* | -0.60 [-0.74, -0.47] | 0.02 [0.00, 0.05] |
|  | *F. damarensis* | -0.60 [-0.73, -0.46] | 0.00 [-0.02, 0.02] |
|  | *F. mechowii* | -0.63 [-0.80, -0.49] | 0.01 [-0.01, 0.04] |
|  | *G. capensis* | -0.58 [-0.70, -0.45] | 0.01 [-0.01, 0.03] |
|  | *H. argenteocinereus* | -0.53 [-0.66, -0.37] | 0.00 [-0.02, 0.03] |
| TJI |  |  |  |
|  | *B. suillus* | -0.63 [-0.80, -0.44] | 0.01 [-0.01, 0.04] |
|  | *C. hottentotus* | -0.71 [-0.88, -0.55] | 0.02 [-0.01, 0.05] |
|  | *F. damarensis* | -0.66 [-0.82, -0.47] | 0.00 [-0.03, 0.03] |
|  | *F. mechowii* | -0.69 [-0.86, -0.51] | 0.01 [-0.02, 0.04] |
|  | *G. capensis* | -0.72 [-0.89, -0.56] | 0.02 [-0.01, 0.04] |
|  | *H. argenteocinereus* | -0.73 [-0.92, -0.56] | 0.02 [0.00, 0.06] |
| IFA |  |  |  |
|  | *B. suillus* | -1.25 [-1.49, -0.94] | -0.03 [-0.07, 0.01] |
|  | *C. hottentotus* | -1.41 [-1.69, -1.17] | 0.04 [0.00, 0.10] |
|  | *F. damarensis* | -1.41 [-1.67, -1.17] | 0.04 [0.00, 0.09] |
|  | *F. mechowii* | -1.29 [-1.53, -0.99] | 0.02 [-0.03, 0.06] |
|  | *G. capensis* | -1.40 [-1.62, -1.17] | 0.02 [-0.01, 0.06] |
|  | *H. argenteocinereus* | -1.31 [-1.53, -1.05] | 0.02 [-0.02, 0.07] |
|  | *H. glaber* | -1.36 [-1.54, -1.16] | 0.02 [-0.01, 0.06] |

**Supplementary Table 5.** Bone superstructures analyzed in this study and their fossorial functional significance. Data obtained from several sources (see Supplementary References 1-23).

| Bone | Character | Morpho-Function | References |
| --- | --- | --- | --- |
| Humerus (Stylopod) | Deltoid tuberosity (DT) | An enlarged, protuberant and distally located DT increases the area for the insertion of *mm. deltoidei* and *mm. pectorales*, and increases the in-lever arm distance from muscle attachment to joint. This produces a powerful stroke (flexion) of the humerus on the scapula and subsequent retraction of the arm during parasagittal scratch-digging. | 1,6,7,9,10,11,13,14,20,21 |
| Ulna (Zeugopod) | Olecranon process (OP) | An enlarged OP increases the area for the insertion of the *mm. triceps brachii,* hence increase the in-lever arm of forearm extensors and enables greater out-forces during scratch-digging. | 4,8,17,22,23 |
| Femur (Stylopod) | Third trochanter (TT) | A mediolaterally enlarged and distally positioned TT increases the insertion area for the *m. gluteus superficialis* for powerful leg extension and some degree of abduction (to brace against a tunnel or pushing back soil while digging), as well as increases medioalteral cortical thicknss of the diaphysis, which reduces bending strains on the femoral shaft due to rearward motion. | 12,16,18 |
| Tibia-Fibula (Zeugopods) | Distal fusion of tibia-fibula (DFTF) | Distal fusion provides a powerful bony base for the attachment of the muscles acting on the paws, as well as increases stabilization of the foot to better withstand load-bearing and bending strains during heavy-impact activities against a resistant medium such as earth. | 2,3,5,6,15,18,19,20 |

**Supplementary Table 6.** Ecological and morpho-functional characteristics of fossorial rodents including 17 genera and 35 species, and the non-fossorial closest relatives of Bathyergidae. All fossorial taxa present a well-developed and projected deltoid tuberosity (DT), except *Heterocephalus glaber,* where such trait is highly reduced. Ecological and morphological data obtained from several sources (see Supplementary References 22,24,28,30,38,40-75).

| Family | Genus | Species | Locomotor mode | Digging mode | Social organization | DT |
| --- | --- | --- | --- | --- | --- | --- |
| Ctenohystrica |  |  |  |  |  |  |
| Bathyergidae | *Heterocephalus* | *glaber* | Fossorial | Chisel-tooth | Highly social | No |
| Bathyergidae | *Heliophobius* | *argenteocinereus* | Fossorial | Chisel-tooth | Solitary | Yes |
| Bathyergidae | *Bathyergus* | *suillus* | Fossorial | Scratch | Solitary | Yes |
| Bathyergidae | *Bathyergus* | *janetta* | Fossorial | Scratch | Solitary | Yes |
| Bathyergidae | *Georychus* | *capensis* | Fossorial | Chisel-tooth | Solitary | Yes |
| Bathyergidae | *Cryptomys* | *hottentotus pretoriae* | Fossorial | Chisel-tooth | Social | Yes |
| Bathyergidae | *Cryptomys* | *hottentotus natalensis* | Fossorial | Chisel-tooth | Social | Yes |
| Bathyergidae | *Cryptomys* | *hottentotus mahali* | Fossorial | Chisel-tooth | Social | Yes |
| Bathyergidae | *Fukomys* | *damarensis* | Fossorial | Chisel-tooth | Highly social | Yes |
| Bathyergidae | *Fukomys* | *mechowii* | Fossorial | Chisel-tooth | Highly social | Yes |
| Hystricidae | *Atherurus* | *macrourus* | Semifossorial | Scratch | Gregarious/Solitary | Yes |
| Hystricidae | *Atherurus* | *africanus* | Ambulatory |  | Solitary | Yes |
| Hystricidae | *Hystrix* | *africaeaustralis* | Semifossorial | Scratch | Gregarious/Social | Yes |
| Hystricidae | *Hystrix* | *cristata* | Semifossorial | Scratch | Gregarious/Solitary | Yes |
| Hystricidae | *Hystrix* | *indica* | Semifossorial | Scratch | Gregarious/Solitary | Yes |
| Petromuridae | *Petromus* | *typicus* | Rock climber |  | Gregarious/Solitary | Yes |
| Thryonomyidae | *Thryonomys* | *swinderianus* | Semiaquatic/Semifossorial | Scratch | Gregarious/Solitary | Yes |
| Ctenomyidae | *Ctenomys* | *flamarioni* | Fossorial | Scratch/Chisel-tooth | Solitary | Yes |
| Ctenomyidae | *Ctenomys* | *lewisi* | Fossorial | Scratch/Chisel-tooth | Solitary | Yes |
| Ctenomyidae | *Ctenomys* | *talarum* | Fossorial | Scratch/Chisel-tooth | Solitary | Yes |
| Ctenomyidae | *Ctenomys* | *rionegrensis* | Fossorial | Scratch/Chisel-tooth | Solitary | Yes |
| Ctenomyidae | *Ctenomys* | *leucodon* | Fossorial | Scratch/Chisel-tooth | Solitary | Yes |
| Ctenomyidae | *Ctenomys* | *minutus* | Fossorial | Scratch/Chisel-tooth | Solitary | Yes |
| Ctenomyidae | *Ctenomys* | *lami* | Fossorial | Scratch/Chisel-tooth | Solitary | Yes |
| Ctenomyidae | *Ctenomys* | *torquatus* | Fossorial | Scratch/Chisel-tooth | Solitary | Yes |
| Ctenomyidae | *Ctenomys* | *fulvus* | Fossorial | Scratch/Chisel-tooth | Solitary | Yes |
| Octodontidae | *Spalacopus* | *cyanus* | Fossorial | Chisel-tooth | Social | Yes |
| Geomyoidea/Myomorpha | |  |  |  |  |  |
| Geomyidae | *Geomys* | *bursarius* | Fossorial | Scratch | Solitary | Yes |
| Geomyidae | *Thomomys* | *bottae* | Fossorial | Chisel-Tooth/Scratch | Solitary | Yes |
| Geomyidae | *Thomomys* | *talpoides* | Fossorial | Chisel-Tooth/Scratch | Solitary | Yes |
| Geomyidae | *Thomomys* | *mazama* | Fossorial | Chisel-Tooth/Scratch | Solitary | Yes |
| Geomyidae | *Thomomys* | *towsendii* | Fossorial | Chisel-Tooth/Scratch | Solitary | Yes |
| Spalacidae | *Myospalax* | *myospalax* | Fossorial | Scratch/Head-Lift | Solitary | Yes |
| Spalacidae | *Rhizomys* | *pruinosus* | Fossorial | Chisel-Tooth/Scratch | Solitary | Yes |
| Spalacidae | *Rhizomys* | *sinensis* | Fossorial | Chisel-Tooth/Scratch | Solitary | Yes |
| Spalacidae | *Rhizomys* | *sumatrensis* | Fossorial | Chisel-Tooth/Scratch | Solitary | Yes |
| Spalacidae | *Spalax* | *ehrenbergi* | Fossorial | Head-Lift/Chisel-tooth | Solitary | Yes |
| Spalacidae | *Spalax* | *microphthalmus* | Fossorial | Head-Lift/Chisel-tooth | Solitary/Gregarious | Yes |
| Spalacidae | *Nannospalax* | *nehringi* | Fossorial | Head-Lift/Chisel-tooth | Solitary | Yes |
| Cricetidae | *Arvicola* | *(terrestri) scherman* | Fossorial | Chisel-tooth | Solitary | Yes |
| Cricetidae | *Ellobius* | *lutescens* | Fossorial | Chisel-tooth | Solitary/Gregarious | Yes |
| Sciuroidea |  |  |  |  |  |  |
| Aplodontidae | *Aplodontia* | *rufa* | Fossorial | Scratch | Solitary/Gregarious | Yes |

**Supplementary Table 7.** Morpho-functional indices and body masses (BM) of individuals analyzed in this study. Body mass in grams (g). Relative position of the deltoid tuberosity (RDT); Index of fossorial ability (IFA); Tibio-fibular junction index (TJI).

| Species | ID | BM (g) | IFA | RDP | TJI |
| --- | --- | --- | --- | --- | --- |
| *B. suillus* | 314 | 998 | 0.246 | 0.596 | 0.595 |
| *B. suillus* | 365 | 804 | 0.266 | 0.594 | 0.584 |
| *B. suillus* | 366 | 748 | 0.237 | 0.620 | 0.584 |
| *B. suillus* | 377 | 918 | 0.223 | 0.583 | 0.583 |
| *B. suillus* | 717 | 1110 | 0.233 | 0.569 | 0.553 |
| *B. suillus* | 721 | 640 | 0.220 | 0.585 | 0.612 |
| *B. suillus* | 911 | 1152 | 0.231 | 0.581 | 0.598 |
| *B. suillus* | 913 | 366 | 0.264 | 0.572 | 0.580 |
| *B. suillus* | 938 | 468 | 0.259 | 0.570 | 0.603 |
| *B. suillus* | 982 | 422 | 0.263 | 0.607 | 0.602 |
| *B. suillus* | 1085 | 778 | 0.253 | 0.612 | 0.616 |
| *B. suillus* | 1138 | 1138 | 0.248 | 0.603 | 0.601 |
| *B. suillus* | 1144 | 914 | 0.236 | 0.584 | 0.606 |
| *B. suillus* | 1153 | 882 | 0.265 | 0.625 | 0.590 |
| *B. suillus* | 1155 | 1066 | 0.240 | 0.572 | 0.621 |
| *B. suillus* | 1163 | 760 | 0.261 | 0.632 | 0.594 |
| *B. suillus* | 1169 | 910 | 0.226 | 0.608 | 0.631 |
| *B. suillus* | 1171 | 1346 | 0.233 | 0.616 | 0.576 |
| *B. suillus* | 1332 | 958 | 0.232 | 0.593 | 0.595 |
| *B. suillus* | 1336 | 508 | 0.258 | 0.578 | 0.603 |
| *B. suillus* | 217 | 1278 | 0.253 | 0.586 | 0.616 |
| *B. suillus* | 220 | 1726 | 0.232 | 0.586 | 0.602 |
| *B. suillus* | 313 | 1072 | 0.243 | 0.585 | 0.557 |
| *B. suillus* | 713 | 1614 | 0.228 | 0.606 | 0.630 |
| *B. suillus* | 765 | 830 | 0.257 | 0.607 | 0.628 |
| *B. suillus* | 861 | 1636 | 0.224 | 0.593 | 0.593 |
| *B. suillus* | 910 | 662 | 0.246 | 0.567 | 0.597 |
| *B. suillus* | 964 | 584 | 0.280 | 0.589 | 0.608 |
| *B. suillus* | 965 | 1228 | 0.251 | 0.582 | 0.589 |
| *B. suillus* | 1039 | 1336 | 0.215 | 0.604 | 0.596 |
| *B. suillus* | 1050 | 1332 | 0.243 | 0.629 | 0.578 |
| *B. suillus* | 1139 | 752 | 0.264 | 0.557 | 0.613 |
| *B. suillus* | 1154 | 780 | 0.271 | 0.601 | 0.628 |
| *B. suillus* | 1338 | 1450 | 0.246 | 0.574 | 0.614 |
| *B. suillus* | 1339 | 788 | 0.252 | 0.597 | 0.620 |
| *B. suillus* | GM282 | 603 | 0.250 | 0.572 | 0.597 |
| *B. suillus* | GM283 | 227 | 0.234 | 0.596 | 0.576 |
| *B. suillus* | GM284 | 1201 | 0.232 | 0.619 | 0.588 |
| *B. suillus* | GM285 | 658 | 0.253 | 0.614 | 0.593 |
| *G.capensis* | NB21 | 141 | 0.280 | 0.581 | 0.554 |
| *G.capensis* | GM530 | 121 | 0.277 | 0.616 | 0.510 |
| *G.capensis* | JO402 | 180 | 0.268 | 0.573 |  |
| *G.capensis* | GM295 | 260 | 0.255 | 0.610 |  |
| *G.capensis* | GM296 | 105 | 0.212 | 0.566 |  |
| *G.capensis* | GM297 | 185 | 0.271 | 0.573 |  |
| *G.capensis* | GM298 | 210 | 0.265 | 0.593 |  |
| *G.capensis* | Z4 | 225 | 0.261 | 0.568 |  |
| *G.capensis* | Z9 | 223.1 | 0.292 | 0.573 |  |
| *G.capensis* | Z10 | 210.2 | 0.286 | 0.594 |  |
| *G.capensis* | Z11 | 105.8 | 0.251 | 0.566 |  |
| *G.capensis* | Z12 | 169.8 | 0.247 | 0.605 |  |
| *G.capensis* | Z22 | 149 | 0.290 | 0.594 |  |
| *G.capensis* | Z23 | 167 | 0.249 | 0.562 |  |
| *G.capensis* | Z25 | 190 | 0.227 | 0.574 |  |
| *G.capensis* | Z27 | 300.8 | 0.305 | 0.581 |  |
| *G.capensis* | Z28 | 171.1 | 0.262 | 0.588 |  |
| *G.capensis* | Z31 | 235.1 | 0.295 | 0.595 |  |
| *G.capensis* | Z32 | 197.4 | 0.292 | 0.614 |  |
| *G.capensis* | Z33 | 126.6 | 0.267 | 0.593 |  |
| *G.capensis* | Z35 | 170.2 | 0.273 | 0.573 |  |
| *G.capensis* | Z36 | 229.5 | 0.318 | 0.587 |  |
| *G.capensis* | Z37 | 135.5 |  | 0.577 |  |
| *G.capensis* | Z38 | 116.6 | 0.238 | 0.552 |  |
| *G.capensis* | Z210 | 141 | 0.274 | 0.562 |  |
| *G.capensis* | Z212 | 156.7 | 0.269 |  |  |
| *G.capensis* | Z214 | 150.8 | 0.279 | 0.557 |  |
| *G.capensis* | Z215 | 298 | 0.245 | 0.536 |  |
| *G.capensis* | Z216 | 175 | 0.244 | 0.563 |  |
| *G.capensis* | Z225 | 75.49 | 0.277 | 0.613 | 0.514 |
| *G.capensis* | A01 | 91.85 | 0.255 | 0.567 | 0.537 |
| *G.capensis* | A03 | 89.21 | 0.269 | 0.566 | 0.492 |
| *G.capensis* | A06 | 95.99 | 0.270 | 0.599 | 0.490 |
| *G.capensis* | A19 | 78.05 | 0.281 | 0.584 | 0.515 |
| *G.capensis* | A35 | 164.72 | 0.270 | 0.572 | 0.485 |
| *G.capensis* | A40 | 166.18 | 0.283 | 0.543 | 0.536 |
| *G.capensis* | A43 | 176.27 | 0.265 | 0.632 | 0.508 |
| *G.capensis* | A51 | 163.39 | 0.292 | 0.581 | 0.507 |
| *G.capensis* | A95 | 175.86 | 0.273 | 0.578 | 0.500 |
| *G.capensis* | A97 | 277.75 | 0.276 | 0.634 | 0.496 |
| *G.capensis* | A99 | 284.02 | 0.281 | 0.587 | 0.522 |
| *G.capensis* | A100 | 274.39 | 0.288 | 0.569 | 0.544 |
| *G.capensis* | A115 | 283.3 | 0.258 | 0.613 | 0.533 |
| *G.capensis* | A118 | 272.63 | 0.263 | 0.595 | 0.537 |
| *G.capensis* | A128 | 177.39 | 0.260 | 0.618 | 0.484 |
| *G.capensis* | A130 | 168.25 | 0.273 | 0.578 | 0.532 |
| *G.capensis* | A131 | 100.42 | 0.281 | 0.606 | 0.481 |
| *H.argenteocinereus* | 526 | 101.4 | 0.311 | 0.631 | 0.530 |
| *H.argenteocinereus* | 26 | 122 | 0.293 | 0.621 | 0.526 |
| *H.argenteocinereus* | 11 | 129 | 0.318 | 0.631 | 0.537 |
| *H.argenteocinereus* | 387 | 130 | 0.307 | 0.599 | 0.487 |
| *H.argenteocinereus* | 27 | 135 | 0.289 | 0.590 | 0.505 |
| *H.argenteocinereus* | 386 | 145 | 0.290 | 0.622 | 0.501 |
| *H.argenteocinereus* | 8 | 149 | 0.283 | 0.639 | 0.508 |
| *H.argenteocinereus* | 6 | 160 | 0.291 | 0.620 | 0.542 |
| *H.argenteocinereus* | 516 | 166.5 | 0.330 | 0.612 | 0.549 |
| *H.argenteocinereus* | 361 | 181 | 0.291 | 0.573 | 0.573 |
| *H.argenteocinereus* | 517 | 190 | 0.301 | 0.627 | 0.583 |
| *H.argenteocinereus* | 585 | 206 | 0.305 | 0.626 | 0.548 |
| *H.argenteocinereus* | 377 | 210 | 0.345 | 0.607 | 0.543 |
| *H.argenteocinereus* | 451 | 235.8 | 0.285 | 0.581 | 0.559 |
| *H.argenteocinereus* | 5 | 249 | 0.303 | 0.601 | 0.455 |
| *H.argenteocinereus* | 18 | 298 | 0.290 | 0.598 | 0.592 |
| *H.argenteocinereus* | 473 | 231 | 0.325 | 0.616 | 0.589 |
| *H.argenteocinereus* | 476 | 137.6 | 0.298 | 0.637 | 0.579 |
| *H.argenteocinereus* | 508 | 225.4 | 0.352 | 0.612 | 0.549 |
| *H.argenteocinereus* | 525 | 112 | 0.297 | 0.598 | 0.539 |
| *H.argenteocinereus* | 492 | 138.4 | 0.318 | 0.625 | 0.548 |
| *H.argenteocinereus* | 479 | 176.2 | 0.293 | 0.648 | 0.550 |
| *H.argenteocinereus* | 244 | 207.5 | 0.303 | 0.600 | 0.528 |
| *H.argenteocinereus* | 590 | 251.5 | 0.326 | 0.607 | 0.517 |
| *H.argenteocinereus* | 241 | 100.2 | 0.298 | 0.597 | 0.497 |
| *H.argenteocinereus* | 242 | 107.3 | 0.317 | 0.611 | 0.480 |
| *H.argenteocinereus* | 243 | 218.24 | 0.281 | 0.630 | 0.509 |
| *H.argenteocinereus* | 244 | 187 | 0.321 | 0.584 | 0.454 |
| *C.hottentotus* | GM102 | 51 | 0.255 | 0.613 | 0.483 |
| *C.hottentotus* | GM103 | 65 | 0.247 | 0.582 | 0.546 |
| *C.hottentotus* | GM104 | 78 | 0.253 | 0.611 | 0.511 |
| *C.hottentotus* | GM105 | 78 | 0.277 | 0.602 | 0.515 |
| *C.hottentotus* | GM106 | 98 | 0.291 | 0.602 | 0.524 |
| *C.hottentotus* | GM108 | 62 | 0.278 | 0.569 | 0.510 |
| *C.hottentotus* | GM109 | 110 | 0.283 | 0.587 | 0.539 |
| *C.hottentotus* | GM110 | 54 | 0.259 | 0.605 | 0.540 |
| *C.hottentotus* | GM111 | 68 | 0.267 | 0.612 | 0.478 |
| *C.hottentotus* | GM112 | 61 | 0.274 | 0.649 | 0.519 |
| *C.hottentotus* | GM114 | 73 | 0.291 | 0.638 | 0.545 |
| *C.hottentotus* | GM115 | 52 | 0.265 | 0.564 | 0.509 |
| *C.hottentotus* | GM116 | 89 | 0.290 | 0.640 | 0.554 |
| *C.hottentotus* | GM117 | 72 | 0.283 | 0.572 | 0.543 |
| *C.hottentotus* | GM122 | 77 | 0.301 | 0.567 | 0.480 |
| *C.hottentotus* | GM123 | 102 | 0.278 | 0.595 | 0.522 |
| *C.hottentotus* | GM126 | 67 | 0.294 | 0.565 | 0.563 |
| *C.hottentotus* | GM127 | 61 | 0.290 | 0.584 | 0.587 |
| *C.hottentotus* | GM128 | 80 | 0.303 | 0.613 | 0.513 |
| *C.hottentotus* | GM129 | 130 | 0.300 | 0.618 | 0.569 |
| *C.hottentotus* | GM136 | 60.53 | 0.293 | 0.581 | 0.474 |
| *C.hottentotus* | GM137 | 76 | 0.275 | 0.570 | 0.509 |
| *C.hottentotus* | GM138 | 84 | 0.296 | 0.595 | 0.524 |
| *C.hottentotus* | GM139 | 53 | 0.300 | 0.588 | 0.539 |
| *C.hottentotus* | GM140 | 54 | 0.270 | 0.564 | 0.527 |
| *C.hottentotus* | GM142 | 47 | 0.276 | 0.561 | 0.555 |
| *C.hottentotus* | GM143 | 57 | 0.256 | 0.571 | 0.532 |
| *C.hottentotus* | GM144 | 71 | 0.305 | 0.594 | 0.528 |
| *C.hottentotus* | GM145 | 92 | 0.260 | 0.552 | 0.561 |
| *C.hottentotus* | GM146 | 50 | 0.250 | 0.607 | 0.510 |
| *C.hottentotus* | GM147 | 68.15 | 0.263 | 0.578 | 0.513 |
| *C.hottentotus* | GM150 | 69 | 0.255 | 0.571 | 0.555 |
| *C.hottentotus* | GM151 | 70 | 0.287 | 0.540 | 0.533 |
| *C.hottentotus* | GM152 | 68 | 0.256 | 0.601 | 0.510 |
| *C.hottentotus* | GM153 | 80 | 0.286 | 0.611 | 0.516 |
| *F.damarensis* | G4F042 | 87 | 0.274 | 0.540 | 0.506 |
| *F.damarensis* | G4M018 | 148 | 0.260 | 0.533 | 0.493 |
| *F.damarensis* | G4M034 | 155 | 0.299 | 0.563 | 0.528 |
| *F.damarensis* | G4M039 | 141 | 0.286 | 0.559 | 0.497 |
| *F.damarensis* | G4M041 | 71 | 0.281 | 0.594 | 0.533 |
| *F.damarensis* | G4M008 | 56 | 0.251 | 0.530 | 0.516 |
| *F.damarensis* | G5F003 | 71 | 0.295 | 0.530 | 0.561 |
| *F.damarensis* | L29F002 | 87 | 0.299 | 0.536 | 0.514 |
| *F.damarensis* | G4F012 | 128 | 0.265 | 0.529 | 0.564 |
| *F.damarensis* | G4F002 | 133 | 0.299 | 0.535 | 0.553 |
| *F.damarensis* | G4M009 | 141 | 0.318 | 0.552 | 0.528 |
| *F.damarensis* | G3M007 | 142 | 0.278 | 0.533 | 0.532 |
| *F.damarensis* | G4M001 | 147 | 0.308 | 0.529 | 0.536 |
| *F.damarensis* | G3M001 | 173 | 0.298 | 0.546 | 0.517 |
| *F.damarensis* | Z3M006 | 226 | 0.305 | 0.545 | 0.534 |
| *F.damarensis* | NB227 | 67.46 | 0.278 | 0.546 | 0.553 |
| *F.damarensis* | NB228 | 63.48 | 0.256 | 0.559 | 0.574 |
| *F.damarensis* | GAP315 | 113 | 0.303 | 0.567 |  |
| *F.mechowii* | 23 | 133 | 0.302 | 0.547 | 0.535 |
| *F.mechowii* | 179 | 139 | 0.325 | 0.545 | 0.529 |
| *F.mechowii* | 3 | 144 | 0.314 | 0.538 | 0.548 |
| *F.mechowii* | 1 | 158 | 0.304 | 0.559 | 0.536 |
| *F.mechowii* | 177 | 232 | 0.278 | 0.570 | 0.578 |
| *F.mechowii* | 497 | 268 | 0.318 | 0.592 | 0.474 |
| *F.mechowii* | 478 | 380.7 | 0.303 | 0.566 | 0.556 |
| *F.mechowii* | 494 | 208.8 | 0.300 | 0.569 | 0.501 |
| *F.mechowii* | 589 | 277.8 | 0.311 | 0.557 | 0.524 |
| *F.mechowii* | 242 | 405.5 | 0.295 | 0.567 | 0.564 |
| *F.mechowii* | 2 | 193.4 | 0.260 | 0.532 | 0.590 |
| *F.mechowii* | 531 | 262.2 | 0.321 | 0.570 | 0.554 |
| *H.glaber* | JJ-001 | 32.29 | 0.245 |  |  |
| *H.glaber* | JJ-002 | 14.96 | 0.246 |  |  |
| *H.glaber* | JJ-045 | 56.38 | 0.256 |  |  |
| *H.glaber* | JJ-046 | 16.41 | 0.265 |  |  |
| *H.glaber* | 1200-047 | 14.9 | 0.246 |  |  |
| *H.glaber* | 1200-048 | 29.84 | 0.251 |  |  |
| *H.glaber* | 1200-049 | 19.82 | 0.288 |  |  |
| *H.glaber* | 1200-050 | 20.82 | 0.253 |  |  |
| *H.glaber* | 1200-051 | 22.41 | 0.296 |  |  |
| *H.glaber* | 1200-052 | 16.37 | 0.267 |  |  |
| *H.glaber* | 1200-053 | 32.14 | 0.281 |  |  |
| *H.glaber* | 1200-054 | 31.5 | 0.267 |  |  |
| *H.glaber* | 1200-055 | 26.82 | 0.260 |  |  |
| *H.glaber* | 1200-056 | 24.027 | 0.222 |  |  |
| *H.glaber* | 1200-057 | 32.47 | 0.269 |  |  |
| *H.glaber* | 1200-058 | 30.24 | 0.279 |  |  |
| *H.glaber* | 1200-059 | 17.72 | 0.279 |  |  |
| *H.glaber* | 1200-060 | 35.81 | 0.297 |  |  |
| *H.glaber* | 1200-061 | 39.91 | 0.247 |  |  |
| *H.glaber* | 1200-063 | 22.85 | 0.280 |  |  |
| *H.glaber* | 1200-064 | 31.29 | 0.278 |  |  |
| *H.glaber* | 1200-065 | 32.71 | 0.287 |  |  |
| *H.glaber* | 1200-066 | 19.08 | 0.261 |  |  |
| *H.glaber* | 1200-067 | 19.72 | 0.282 |  |  |
| *H.glaber* | 1200-068 | 23.45 |  |  |  |
| *H.glaber* | 1200-069 | 15.29 | 0.300 |  |  |
| *H.glaber* | 1000-070 | 31.94 | 0.275 |  |  |
| *H.glaber* | 1000-071 | 34.58 | 0.273 |  |  |
| *H.glaber* | 1000-072 | 26.77 | 0.268 |  |  |
| *H.glaber* | 1000-073 | 28.56 | 0.281 |  |  |
| *H.glaber* | 1000-074 | 26.44 | 0.291 |  |  |
| *H.glaber* | 1000-075 | 23.95 | 0.262 |  |  |
| *H.glaber* | 1000-076 | 29.54 | 0.258 |  |  |
| *H.glaber* | 1000-077 | 44.58 | 0.320 |  |  |
| *H.glaber* | 1000-078 | 36.08 | 0.255 |  |  |
| *H.glaber* | 1000-079 | 23.88 | 0.271 |  |  |
| *H.glaber* | 1000-080 | 41.44 | 0.294 |  |  |
| *H.glaber* | 1000-081 | 34.45 | 0.281 |  |  |
| *H.glaber* | 1000-082 | 43.97 | 0.302 |  |  |
| *H.glaber* | 1000-083 | 23.43 | 0.289 |  |  |
| *H.glaber* | 1000-084 | 34.49 | 0.263 |  |  |
| *H.glaber* | 1000-085 | 27.19 | 0.254 |  |  |
| *H.glaber* | 1000-086 | 28.72 | 0.275 |  |  |
| *H.glaber* | 5000-087 | 31.06 | 0.266 |  |  |
| *H.glaber* | 5000-088 | 22.57 | 0.264 |  |  |
| *H.glaber* | 5000-089 | 22.3 | 0.277 |  |  |
| *H.glaber* | 5000-090 | 35.17 | 0.297 |  |  |
| *H.glaber* | 5000-091 | 28.51 | 0.259 |  |  |
| *H.glaber* | 5000-092 | 34.4 | 0.293 |  |  |
| *H.glaber* | 5000-093 | 30.4 | 0.258 |  |  |
| *H.glaber* | 5000-094 | 33.83 | 0.275 |  |  |
| *H.glaber* | 5000-095 | 28.86 | 0.261 |  |  |
| *H.glaber* | 5000-096 | 24.63 | 0.215 |  |  |
| *H.glaber* | 5000-097 | 31.13 | 0.292 |  |  |
| *H.glaber* | 5000-098 | 29.36 | 0.276 |  |  |
| *H.glaber* | 5000-099 | 29.82 | 0.273 |  |  |
| *H.glaber* | 5000-100 | 21.64 | 0.297 |  |  |
| *H.glaber* | 5000-101 | 19.21 | 0.261 |  |  |
| *H.glaber* | GM420 | 22.92 | 0.264 |  |  |
| *H.glaber* | GM498 | 47 | 0.247 |  |  |
| *H.glaber* | GM499 | 66 | 0.293 |  |  |
| *H.glaber* | GM500 | 17.93 | 0.259 |  |  |
| *H.glaber* | GM501 | 63 | 0.265 |  |  |
| *H.glaber* | GM505 | 19.58 | 0.282 |  |  |
| *H.glaber* | GM506 | 16.04 | 0.263 |  |  |
| *H.glaber* | GM507 | 41 | 0.270 |  |  |
| *H.glaber* | GM508 | 35 | 0.245 |  |  |
| *H.glaber* | GM509 | 39 | 0.272 |  |  |
| *H.glaber* | GM510 | 30 | 0.244 |  |  |
| *H.glaber* | GM511 | 33 | 0.257 |  |  |

**SUPPLEMENTARY FIGURES**

**Supplementary Figure 1.** Humeral phenotype of African mole-rats analyzed here. a) Posterior view. b) Lateral view. Bones ordered same as in “a”. Abbreviations: anterior (a); deltoid tuberosity (DT); dorsal (d), greater tubercle (GTu); humeral head (HH); medial (m).


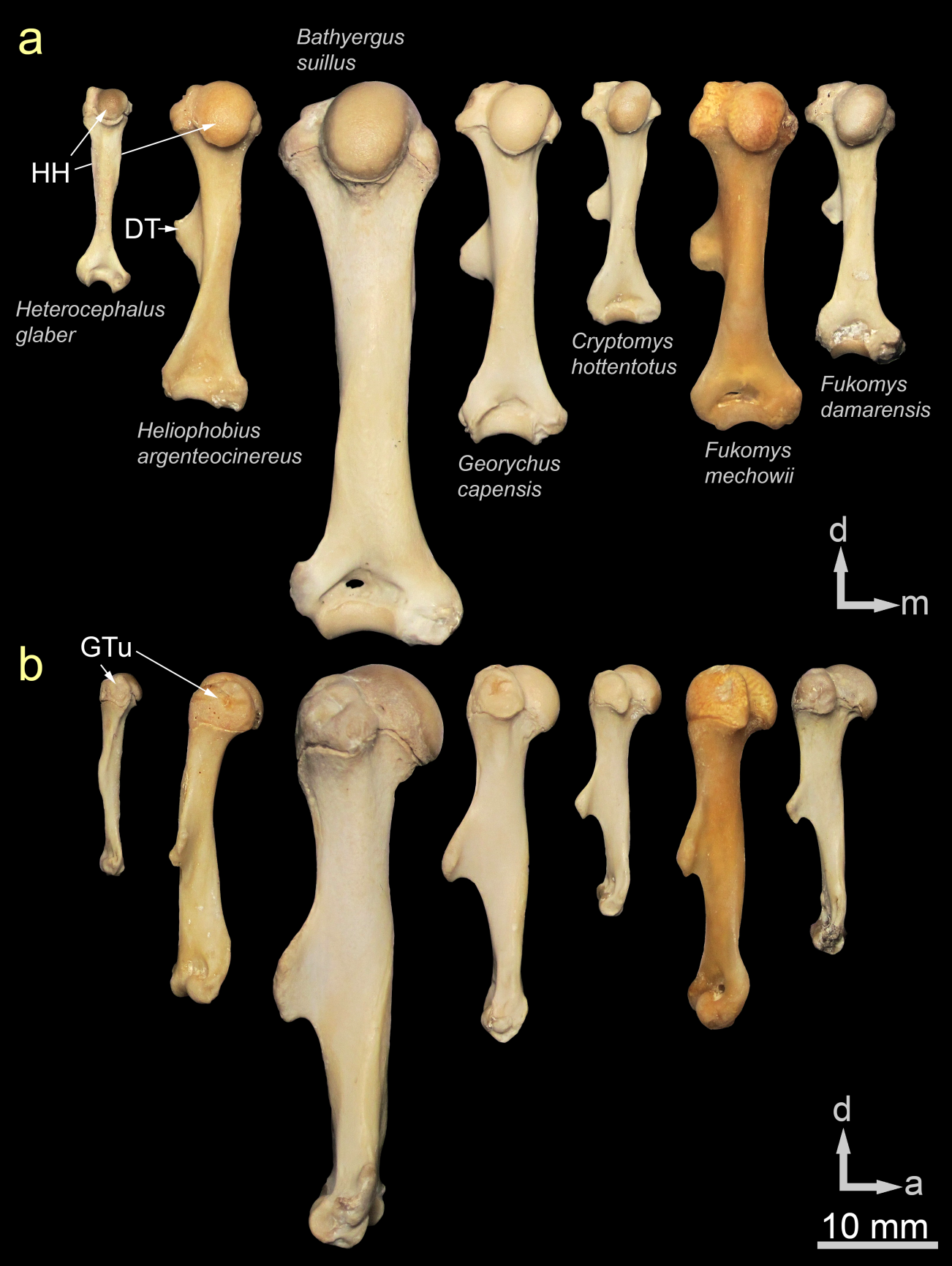


**SUPPLEMENTARY METHODS**

**Ontogenetic stages**

Individuals were classified as newborns, pups, and adults based on chronological age, patterns of molar eruption and tooth wear, and degree of skeletal development (Supplementary Table 1). The ontogeny of *H. argenteocinereus* and *F. mechowii* was based on chronological age. Gomes Rodrigues et al. (76) described that the third molar of *H. argenteocinereus* erupts after the second month of life and the full cheek teeth (four molars) are already developed at two years old, so that individuals older than two years in this study were considered adults and assumed to have developed the fourth molar. Similar classification was used for *F. mechowii*. Newborns are one day old and pups are a few days old. For the rest of the species, individuals having full eruption of all upper or lower molars were considered adults. Almost all genera have four molars (77-80), although *Heterocephalus* has three (sometimes two) (11,81) and some populations of *Heliophobius* *argenteocinereus* exhibit 4-7 molars and continuous dental replacement (76,82,83). Determination of molar eruption in *Bathyergus* was based on Hart et al. (79) and Montoya-Sanhueza et al. (84): age-classes 5-9 are considered as adults. The classification of *F. damarensis* was based on patterns of molar eruption described for *Fukomys mechowii*: age-classes 5-9 were considered as adults (80). In *Cryptomys hottentotus*, individuals of age-class 3-4 are considered as adults (78). Individuals of *H. glaber* with full molar eruption were considered adults (85). For younger ontogenetic stages (pups), other anatomical features indicating skeletal immaturity were used, including poor development of secondary centers of ossification, presence of chondroepiphyses and unfused distal epiphysis in the humerus (84). Perinatal individuals of unknown age were determined as newborns/pups based on their very small size and incipient skeletal development.

**Bone superstructures**

Presence/absence of bone superstructures are presented in Supplementary Table 1. Among the bone superstructures analyzed, the projected and distally located DT is one of the most extensively studied adaptations among fossorial animals, which is principally adapted to accommodate enlarged pectoral and deltoid muscles to increase the power-stroke (retraction) of the arm (Supplementary Table 5). This structure is often referred to in multiple ways, such as tubercle, crest, ridge or process, although proper definition of such terms and the differences among them are rather ambiguous. We define the deltoid tuberosity (= tubercle/process) as a localized and conspicuous protrusion of bone projecting from the diaphysis, with a variable location and size in the diaphysis among mammals. The DT differs from a deltoid crest (DC) (= ridge), which represents an extended bony surface along the diaphysis associated with a wide muscular attachment, but usually not forming a localized protuberance. The DC is observed in some taxa such as tubulidentates, hystricids, tenrecids and solenodontids (34,86). Such structures are not mutually exclusive and a DC can appear at the proximal region of the diaphysis (below the greater tubercle) and extend towards the middle part of the diaphysis fusing with the DT around the midshaft (24), as occurs in hystricids. Two conditions for the DT were recorded in this study: i) the humerus has a smooth diaphyseal surface lacking a projected DT (but not precluding of showing a reduced DT, a small scar for the attachment of the *mm. deltoidei* or a DC); and ii) the humerus has a diaphysis with a conspicuously localized and projected DT.

Regarding the DFTFi, this character has typically been associated with fossoriality, although it is not exclusive to fossorial taxa. Barnett and Napier (19) analyzed the type of tibio-fibular articulation of a wide variety of mammals and found that the fibula of fossorial and aquatic mammals is immobile and united to the tibia at its upper and lower ends by bone (or fibrous) tissue, thus conferring increased robustness and rigidness (Supplementary Table 5). Two conditions were measured: i) tibia and fibula are not distally fused (non-ossified); and ii) tibia and fibula are distally fused and ossified.

Regarding the OP and TT, these features are easily recognizable bony projections, the first located at the proximal ulna, beginning at the base of the anconeal process, and the second located in the proximal femur between the greater trochanter and the midshaft region. Two conditions for these features were recorded, presence or absence, regardless of their cartilaginous or ossified tissue condition.

**Morpho-functional indices**

Seven linear measurements were used to calculate morpho-functional indices: total lengths of humerus (HL), ulna (UL) and tibia-fibula (TL); length of the deltoid tuberosity (DLH); length of the olecranon process (OL), functional length of the ulna (FUL) and length of the distal tibio-fibular junction (DTFJ). Total bone lengths are the maximum distance from the proximal articular surface to the distal articular surface. OL is the length from the tip of the olecranon to the center of the trochlear notch. FUL is the difference between UL and OL. DLH and DTFJ were measured from the proximal articular surface of the bone to the distal origin of the deltoid tuberosity and to the tibio-fibular junction, respectively. All measurements were recorded to the nearest 0.01 mm using a digital caliper. The relative position of the deltoid tuberosity (RDT = DLH/HL) and the index of fossorial ability (IFA = OL/FUL) were calculated following a previous study and references therein (84) (Supplementary Table 7). The tibio-fibular junction index (TJI = DTFJ/TL) is a novel ecomorphological index modified from Montoya-Sanhueza et al. (84) and implemented for the first time in this study to reflect the extension of the distal tibio-fibular fusion, so that lower values (i.e. a more proximal and larger fusion of the tibia and fibula) would suggest a longer bony base and more robust bone diaphysis to increase bone resistance to muscles acting on the paws.

**Phylogenetic varying effects regression**

The amount of change in a given index, for example RDT, in response to a one unit increase in BM for individual *i* belonging to species *j*, was modelled as:

${logRDT}_{i}\sim Normal\left( \mu_{i},\sigma\right)$

$\mu_{i}=\alpha_{j\left[ i \right]}+\beta_{j\left[ i \right]}{logBM}_{i}+\varepsilon_{j\left[ i \right]}$

$\alpha_{j}\sim MVNormal\left( \mu_{\alpha},S_{\alpha} \right)$

$S_{\alpha}=\sigma_{phy_{\alpha}}R$

$\beta_{j}\sim MVNormal\left( \mu_{\beta},S_{\beta} \right)$

$S_{\beta}=\sigma_{phy_{\beta}}R$

$\varepsilon_{j}\sim Normal\left( 0,\sigma_{spp} \right)$

with intercepts α_j_, i.e. species mean phenotypes, and slopes β_j_, i.e. species scaling effects, drawn from multivariate Gaussian distributions, where µ_α_ and µ_β_ are intercept mean and slope mean, and S_α_ and S_β_ are covariance matrices for species’ intercepts and slopes, respectively. Covariance in both intercepts and slopes was defined as the product of a phylogenetic correlation matrix R, with expected correlations among species determined by an Ornstein-Uhlenbeck model of trait evolution (87,88), and variance parameter σ_phy_, giving the magnitude of phylogenetic effect (89). Species-specific effects were captured by a vector of residuals, where each species j had its own residual ε_j_, modeled as a normally distributed random variable with mean zero and standard deviation σ_spp_, giving an overall magnitude of species-specific effects, or in other words, the variation unaccounted for by the phylogenetic component (90,91).

We assigned regularizing priors to all sampled parameters, which reduce the risk of overfitting the data and give more accurate predictions (92). Priors for the sampled parameters were drawn from the following distributions:

$\mu_{\alpha}\sim Normal\left( 0,0.5 \right)$

$\mu_{\beta}\sim Normal\left( 0,0.5 \right)$

$\sigma\sim Exponential(2)$

$\sigma_{phy_{\alpha}}\sim Exponential\left( 2 \right)$

$\sigma_{phy_{\beta}}\sim Exponential\left( 2 \right)$

$\sigma_{spp}\sim Exponential\left( 2 \right)$

**SUPPLEMENTARY REFERENCES**

1. Caspar, K.R, H. Burda, & S. Begall. *Fukomys mechowii* (Rodentia: Bathyergidae), Mammalian Species 53(1011): 145–159 (2021).
2. Bennett, NC. *Cryptomys hottentotus* Common Mole-Rat in *Mammals of Africa: Volume III* (ed. Happold, D. C. D.) 659-660 (Bloomsbury Publishing, 2013).
3. Bennett & Burda. *Cryptomys mechowi* Giant Mole-Rat in *Mammals of Africa: Volume III* (ed. Happold, D. C. D) 659-660 (Bloomsbury Publishing, 2013).
4. Bennett, N.C. & Jarvis, J.U.M. The reproductive biology of the Cape mole‐rat, *Georychus* *capensis* (Rodentia, Bathyergidae). Journal of Zoology, 214: 95-106 (1988).
5. Bennett, N.C. The social structure and reproductive biology of the common mole‐rat, *Cryptomys h. hottentotus* and remarks on the trends in reproduction and sociality in the family Bathyergidae. Journal of Zoology, 219: 45-59 (1989).
6. Brett, R. A. The population structure of naked mole-rat colonies in *The biology of the naked mole-rat* (eds. P. W. Sherman, J. U. M. Jarvis, & R. D. Alexander) 97-136 (Princeton University Press, 1991).
7. Burda, H., R. L. Honeycutt, S. Begall, O. Grütjen, & A. Scharff. Are naked and common mole-rats eusocial and if so, why? Behavioral Ecology and Sociobiology 47:293–303 (2000).
8. Burda H, & Kawalika M. Evolution of eusociality in the Bathyergidae: the case of the giant mole-rats. *Cryptomys mechowi*. Naturwissenschaften 80:235–237 (1993).
9. Herbst, M. The biology and population ecology of the Namaqua dune molerat, *Bathyergus* *janetta* from the Northern Cape Province, South Africa. MSc thesis, University of Pretoria, South Africa (2002).
10. Jarvis, JUM. *Bathyergus suillus* Cape Dune Mole-Rat in *Mammals of Africa: Volume III* (ed. Happold, D. C. D.) 646-648 (Bloomsbury Publishing, 2013).
11. Jarvis JUM., & PW. Sherman. *Heterocephalus glaber*. Mammalian Species, No. 706: 1-9 (2002).
12. Jarvis, J. U. M. Eusociality in a mammal: Cooperative breeding in naked mole-rat colonies. Science 212:571-573 (1981).
13. Jarvis JUM, O’Riain MJ, Bennett NC, & Sherman PW. Mammalian eusociality: a family affair. Trends Ecol Evol 9:47–51 (1994).
14. Jarvis JUM, Bennett NC Eusociality has evolved independently in two genera of bathyergid mole-rats – but occurs in no other subterranean mammal. Behav Ecol Sociobiol 33:253–260 (1993).
15. Kawalika M., Burda H. Giant Mole-rats, *Fukomys mechowii*, 13 Years on the Stage in *Subterranean Rodents* (eds. Begall S., Burda H., Schleich C.E.) (Springer, 2007).
16. Scharff A, O. Locker-Grütjen, M. Kawalika, & H. Burda. Natural History of the Giant Mole-Rat, *Cryptomys* *mechowi* (Rodentia: Bathyergidae), from Zambia. Journal of Mammalogy, 82(4): 1003–1015 (2001).
17. Šumbera, R., Burda, H., Chitaukali, W.N. Reproductive biology of a solitary subterranean bathyergid rodent, the silvery mole-rat (*Heliophobius argenteocinereus*). J. Mammal. 84, 278–287 (2003).
18. Alvarez GI, Díaz AO, Longo MV, Becerra F, & Vassallo AI. Histochemical and morphometric analyses of the musculature of the forelimb of the subterranean rodent Ctenomys talarum (Octodontoidea). Anat Histol Embryol; 41(5):317-25 (2012).
19. Barnett CH, & Napier JR. The rotatory mobility of the fibula in eutherian mammals. J Anat.; 87(1):11-21 (1953).
20. Carleton, A. A comparative study of the inferior tibio-fibular joint. J. Anat., Lond., 76, 45-55 (1941).
21. Fernández ME, Vassallo AI, & Zárate M. Functional morphology and paleobiology of the Pliocene rodent Actenomys (Caviomorpha: Octodontidae): the evolution to a subterranean mode of life. Biological Journal of the Linnean Society 71: 71–90 (2000).
22. Hildebrand M. Digging of quadrupeds In *Functional Vertebrate Morphology* (eds. Hildebrand M, Bramble D, Liem K, & Wake DB) 89–109 (The Belknap Press of Harvard University Press, 1985).
23. Hildebrand M. Insertions and functions of certain flexor muscles in the hind leg of rodents. Journal of Morphology 155(1):111-122 (1978).
24. Holliger, C.D. Anatomical adaptations in the thoracic limb of the California pocket gopher and other rodents. Univ. California Publ. Zool., vol. 13, pp. 447-495 (1916).
25. Goldstein B. Allometric Analysis of Relative Humerus Width and Olecranon Length in Some Unspecialized Burrowing Mammals. Journal of Mammalogy, 53(1): 148-156 (1972).
26. Lagaria A, & Youlatos D. Anatomical correlates to scratch digging in the forelimb of european ground squirrels (Spermophilus citellus). Journal of Mammalogy 87: 563–570 (2006).
27. Lessa EP, & Stein BR. Morphological constraints in the digging apparatus of pocket gophers (Mammalia, Geomyidae). Biological Journal of the Linnean Society 47: 439–453 (1992).
28. Lessa EP, Vassallo AI, Verzi DH, & Mora MS. Evolution of morphological adaptations for digging in living and extinct ctenomyid and octodontid rodents. Biological Journal of the Linnean Society 95: 267–283 (2008).
29. Milne N, & O'Higgins P. Scaling of form and function in the xenarthran femur: a 100-fold increase in body mass is mitigated by repositioning of the third trochanter. Proc Biol Sci. 7; 279(1742):3449-56 (2012).
30. Morgan CC, & Verzi DH. Morphological diversity of the humerus of the South American subterranean rodent Ctenomys (Rodentia, Ctenomyidae). Journal of Mammalogy 87: 1252–1260 (2006).
31. Morgan, C.C. & Álvarez, A. Shape variation of humerus of caviomorph rodents. J Zool, 290: 107-116 (2013).
32. Parsons, F. G. Myology of rodents. Part 11, an account of the myology of the Myomorpha, together with a comparison of the muscles of various suborders of rodents. Proc. zool. SOC. Lund. 1896: 159-192 (1896).
33. Samuels JX, & Valkenburgh B Van. Skeletal Indicators of Locomotor Adaptations in Living and Extinct Rodents. Journal of Morphology 269: 1387–1411 (2008).
34. Salton JA, & Sargis EJ. Evolutionary Morphology of the Tenrecoidea (Mammalia) Forelimb Skeleton. In: Sargis E, Dagosto M, eds. Mammalian Evolutionary Morphology, A Tribute to Frederick S. Szalay. Dordrecht: Springer Netherlands, 51–72 (2008).
35. Salton, J.A. & Sargis, E.J. Evolutionary morphology of the Tenrecoidea (Mammalia) hindlimb skeleton. J. Morphol., 270: 367-387 (2009).
36. Silva MJ, Brodt MD, & Hucker WJ. Finite element analysis of the mouse tibia: estimating endocortical strain during three‐point bending in SAMP6 osteoporotic mice. Anat Rec 283, 380– 390 (2005).
37. Stein B. Morphology of Subterranean Rodents in *Life Underground: the biology of subterranean rodents* (eds. Lacey EA, Patton J, Cameron GN) 19–61 (The University of Chicago Press, 2000).
38. Steiner-Souza, F., De Freitas, T.R.O. & Cordeiro‐Estrela, P. Inferring adaptation within shape diversity of the humerus of subterranean rodent *Ctenomys*. Biological Journal of the Linnean Society, 100: 353-367 (2010).
39. Vassallo AI. Functional morphology, comparative behaviour, and adaptation in two sympatric subterranean rodents genus Ctenomys (Caviomorpha: Octodontidae). J Zool, 244:415–427 (1998).
40. Vizcaíno SF, Fariña RA, & Mazzetta G V. Ulnar dimensions and fossoriality in armadillos. Acta Theriologica 44: 309–320 (1999).
41. Arjo W.M. 2007. Mountain Beaver: A Primitive Fossorial Rodent. In: Begall S., Burda H., Schleich C.E. (eds) Subterranean Rodents. Springer, Berlin, Heidelberg.
42. Begall, S., Burda, H., & M. H. Gallardo. Reproduction, postnatal development and growth of social coruros, *Spalacopus cyanus* (Octodontidae, Rodentia) from Chile. Journal of Mammalogy 80:210–217 (1999).
43. Blumstein, D. Chapter 27, The Evolution of Alarm Communication in Rodents: Structure, Function, and the Puzzle of Apparently Altruistic Calling in *Rodent Societies* (eds. Wolff, J. and Sherman, P.) 317-327 (University of Chicago Press, 2008).
44. Corti M., Fadda C., Simson S., & Nevo E. Size and Shape Variation in the Mandible of the Fossorial Rodent *Spalax ehrenbergi* in *Advances in Morphometrics. NATO ASI Series (Series A: Life Sciences)* vol 284 (eds. Marcus L.F., Corti M., Loy A., Naylor G.J.P., Slice D.E.) (Springer, 1996).
45. Coşkun, Y. On distribution, morphology and biology of the Mole Vole, *Ellobius lutescens* Thomas, 1897 (Mammalia: Rodentia) in eastern Turkey. Zoology in the Middle East, 23(1), 5–12 (2001).
46. Durão, AF, Muñoz‐Muñoz, F, & Ventura, J. Three‐dimensional geometric morphometric analysis of the humerus: Comparative postweaning ontogeny between fossorial and semiaquatic water voles (*Arvicola*). Journal of Morphology; 281: 1679– 1692 (2020).
47. Echeverría AI, Becerra F, & Vassallo A. Postnatal Ontogeny of Limb Proportions and Functional Indices in the Subterranean Rodent *Ctenomys talarum* (Rodentia: Ctenomyidae). Journal of Morphology 275: 902–913 (2014).
48. El-Shafey, A.A, Akraiem A, & A.S.A Abdel-Galil. Radiological Investigation of the African Brush-tailed Porcupine (*Atherurus africanus*) Appendicular Skeleton. 1^st^ Scientific conference Omar ElMokhtar University, 1-15 (2010).
49. Faulkes, C. & Bennett, N. Chapter 36, African Mole-Rats: Social and Ecological Diversity in *Rodent Societies* (eds. J. Wolff & P. Sherman) 427-437 (University of Chicago Press, 2008).
50. Gambaryan, P.P. & Kielan-Jaworowska, Z. Sprawling versus parasagittal stance in multituberculate mammals. - Acta Palaeontolosica Polonica 42, l,13-44 (1997).
51. Gambaryan, P.P. & Gasc J.P. Adaptive properties of the musculoskeletal system in the mole-rat *Myospalax* (Marnmalia, Rodentia), cinefluorographical, anatomical and biomechanical analyses of the burrowing. Zoologische Jahrbuch, Anatomie 123, 363401 (1993).
52. Gambaryan, P.P. Prisposobitel'nie osobennosti organov dvigenia rojuschich mlekopitajuschich (*Adaptive features of locomotion organs in burrowing mammals*). Yerevan, Akad. Nauk Armenian SSR Publ. Office, 195 p. (1960) (In Russian).
53. Gambaryan P.P. Adaptive peculiarities of the forelimb in mole rat (*Spalax leucodon nehringi* Satunin) Zoologicheskii Sbornik AN Armyanskoi SSR. 8:67–125 (1953) (in Russian).
54. Hedrick, B.P. *et al*. The evolutionary diversity of locomotor innovation in rodents is not linked to proximal limb morphology. Sci Rep 10, 717. (2020)
55. Kubiak BB, *et al*. Evolution in action: soil hardness influences morphology in a subterranean rodent (Rodentia: Ctenomyidae). Biol J Linn Soc 20:1–11 (2018).
56. Lacey, EA. Spatial and Social Systems of Subterranean Rodents (Ch. 7.) in *Life Underground: the biology of subterranean rodents* (eds. Lacey EA, Patton J, & Cameron GN) 257-296 (The University of Chicago Press, 2000).
57. Lewis TH. The morphology of the pectoral girdle and anterior limb in *Aplodontia*. J Morphol.; 85(3): 533-558 (1949).
58. Lyon, MW Jr. Notes on the porcupines of the Malay Peninsula and Archipelago. Proceedings of the United States National Museum. 32 (1552):575–594, 4 pls (1907).
59. Marcy AE, Hadly EA, Sherratt E, Garland K, & Weisbecker V. Getting a head in hard soils: Convergent skull evolution and divergent allometric patterns explain shape variation in a highly diverse genus of pocket gophers (*Thomomys*). BMC Evolutionary Biology: 1–16 (2016).
60. Molur, S. *Atherurus macrourus* (amended version of 2016 assessment). The IUCN Red List of Threatened Species 2020: e.T2354A166518819. https://dx.doi.org/10.2305/IUCN.UK.2020-1.RLTS.T2354A166518819.en. Downloaded on 22 March 2021.
61. Monadjem, A., Taylor, P., Denys, C. & Cotterill, F. Rodents of Sub-Saharan Africa. Berlin, München, Boston: De Gruyter. 1092 pp (2015).
62. Morgan CC, Verzi DH, Olivares AI, & Vieytes EC. Craniodental and forelimb specializations for digging in the South American subterranean rodent *Ctenomys* (Hystricomorpha, Ctenomyidae). Mamm Biol 87:118–124 (2017).
63. Morgan CC. The postcranial skeleton of caviomorphs: morphological diversity, adaptations and patterns. Ch 5. SAREM Series A - Mammalogical Research, Vol 1: 167-198 (2015).
64. Nevo, E. Chapter 25. Evolution of Pacifism and Sociality in Blind Mole-Rats in *Rodent Societies* (eds. Wolff, J. & Sherman, P) 291-302 (University of Chicago Press, 2008).
65. Nevo E. Adaptive convergence and divergence of subterranean mammals. Annu Rev Ecol Evol Syst, 10:269–308 (1979).
66. Orcutt E E. Studies on the muscles of the head, neck and pectoral appendages of *Geomys bursarius*. J. Mamm., 21(1): 37-52 (1940).
67. Özkan, ZE. Macro-anatomical investigations on the hind limb skeleton of mole-rat (*Spalax leucodon* Nordmann). Veterinarski Arhiv 72 (2), 91-99 (2002).
68. Pérez, MJ; & Díaz, MM. Postcranial skeleton of *Spalacopus cyanus* (Rodentia: Octodontidae): description and functional aspects; Asociación Mexicana de Mastozoología; Therya; 11; 3; 407-422 (2020).
69. Puzachenko, A. Social organization in the mole rat population, *Spalax microphthalmus* (Rodentia, Spalacidae). Zoologicheskiĭ Zhurnal 72(5):123-131 (1993).
70. Skinner J, & Chimimba CT. The mammals of the Southern African Region. Cape Town, South Africa: Cambridge University Press (2005).
71. Stein, B. R. Phylogenetic relationships among four arvicolid genera. Zeitschrift fur Sdugetierkunde 52: 140-156 (1987).
72. Tobechukwu OK, Adeniyi OS, Olajide HJ, Tavershima D, & Sulaiman SO. Macro–anatomical and morphometric studies of the Grasscutter (*Thryonomyss winderianus*) forelimb skeleton. Int J Vet Sci Anim Husb, 2, 1, 6-12 (2015).
73. Topachevskii, VA. Fauna of the USSR: mammals. Mole rats, Spalacidae. New Delhi: Amerind Publishers. 308 pp (1976).
74. Ventura J, & Götzens V. Prevalence of anomalies in the appendicular skeleton of a fossorial rodent population. J Wildl Dis.; 41(4):728-734 (2005).
75. Yilmaz, S., Z. E. Özkan & D. Özdemir. Oklu Kirpi (*Hystrix cristata*) iskelet Sistemi Uzerinde Makro-Anatomik Araştinnalar. I. Ossa Membri Thoracici. Tr. J. Vet. Anim. Sci. 22:289-392 (1998).
76. Gomes Rodrigues H, *et al*. Continuous dental replacement in a hyper-chisel tooth digging rodent. Proceedings of the National Academy of Sciences of the United States of America 108:17355–17359 (2011).
77. Taylor P, Jarvis J, Crowe T, Davies KC. Age determination in the Cape molerat *Georhychus capensis*. S.-Afr. Tydskr. Dierk. 20: 261–267 (1985).
78. Bennett, N. C., Jarvis, J. U. M. & Wallace, D. B. The relative age structure and body masses of complete wild‐captured colonies of two social mole‐rats, the common mole‐rat, *Cryptomys hottentotus* *hottentotus* and the Damaraland mole‐rat, *Cryptomys damarensis*. J. Zool. 220, 469–485 (1990).
79. Hart L, Chimimba CT, Jarvis JUM, O’Riain J, & Bennett NC. Craniometric Sexual Dimorphism and Age Variation in the South African Cape Dune Mole-Rat (*Bathyergus suillus*). Journal of Mammalogy 88: 657–666 (2007).
80. Chimimba, C.T., A.M. Sichilima, C.G. Faulkes & N.C. Bennett. Ontogenetic variation and craniometric sexual dimorphism in the social giant mole-rat, *Fukomys mechowii* (Rodentia: Bathyergidae), from Zambia, African Zoology, 45:2, 160-176 (2010).
81. Hamilton, W J Jr. *Heterocephalus*, the Remarkable African Burrowing Rodent. The museum of the brooklyn institute of arts and sciences, Vol 3(5) (1928).
82. Gomes Rodrigues H, & Sumbera, R. Dental peculiarities in the silvery mole-rat: an original model for studying the evolutionary and biological origins of continuous dental generation in mammals. PeerJ 3:e1233; DOI 10.7717/peerj.1233 (2015).
83. Katandukila J. Craniometrics analysis for ontogenetic physiognomy and sexual dimorphism in Emin’s silvery Mole-Rats (*Heliophobius argenteocinereus emini*: Bathyergidae) from Tanzania. Tanz J Sci 46 (3):647-660 (2020).
84. Montoya-Sanhueza, G, Wilson LAB & Chinsamy A. Postnatal development of the largest subterranean mammal (*Bathyergus suillus*): Morphology, osteogenesis, and modularity of the appendicular skeleton. Developmental Dynamics. 1-28. DOI: 10.1002/dvdy.81 (2019).
85. Montoya‐Sanhueza, G, Bennett, NC, Oosthuizen, MK, Dengler‐Crish, CM, & Chinsamy, A. Long bone histomorphogenesis of the naked mole‐rat: histodiversity and intraspecific variation. J Anat.: 1– 25 (2021).
86. Lehmann T, Vignaud P, Likius A, Mackaye HT, & Brunet M. A sub-complete fossil aardvark (Mammalia, Tubulidentata) from the Upper Miocene of Chad. Comptes Rendus Palevol 5(5):693–703 (2006).
87. Martins, E. P., & Hansen, T. F. Phylogenies and the comparative method: a general approach to incorporating phylogenetic information into the analysis of interspecific data. The American Naturalist, 149(4), 646-667 (1997).
88. Butler, M. A., & King, A. A. Phylogenetic comparative analysis: a modeling approach for adaptive evolution. The American Naturalist, 164(6), 683-695 (2004).
89. Ives, A. R., & Helmus, M. R. Generalized linear mixed models for phylogenetic analyses of community structure. Ecological Monographs, 81(3), 511-525 (2011).
90. Lynch, M. Methods for the analysis of comparative data in evolutionary biology. Evolution, 45(5), 1065-1080 (1991).
91. Gallinat, A. S., & Pearse, W. D. Phylogenetic generalized linear mixed modeling presents novel opportunities for eco‐evolutionary synthesis. Oikos, 130(5), 669-679 (2021).
92. McElreath, R. Statistical rethinking: A Bayesian course with examples in R and Stan. In Statistical Rethinking: A Bayesian Course with Examples in R and Stan. CRC press (2020).
