# Supplementary material for: Fossorial adaptations in African mole-rats (Bathyergidae) and the unique appendicular phenotype of naked mole-rats
Source: Commun Biol. 2022 Jun 1;5:526. doi: 10.1038/s42003-022-03480-z (PMC9159980; doi:10.1038/s42003-022-03480-z)
Supplement: Supplementary file 3 — Reporting Summary [file 42003_2022_3480_MOESM3_ESM.pdf]

nature portfolio

Corresponding author(s): GERMAN MONTOYA-SANHUEZA

Last updated by author(s): May 2, 2022

Reporting Summary

Nature Portfolio wishes to improve the reproducibility of the work that we publish. This form provides structure for consistency and transparency in reporting. For further information on Nature Portfolio policies, see our Editorial Policies and the Editorial Policy Checklist.

Statistics

For all statistical analyses, confirm that the following items are present in the figure legend, table legend, main text, or Methods section.

n/a	Confirmed	
☐	☒	The exact sample size (n) for each experimental group/condition, given as a discrete number and unit of measurement
☐	☒	A statement on whether measurements were taken from distinct samples or whether the same sample was measured repeatedly
☐	☒	The statistical test(s) used AND whether they are one- or two-sided<br>*Only common tests should be described solely by name; describe more complex techniques in the Methods section.*
☐	☒	A description of all covariates tested
☐	☒	A description of any assumptions or corrections, such as tests of normality and adjustment for multiple comparisons
☐	☒	A full description of the statistical parameters including central tendency (e.g. means) or other basic estimates (e.g. regression coefficient) AND variation (e.g. standard deviation) or associated estimates of uncertainty (e.g. confidence intervals)
☐	☒	For null hypothesis testing, the test statistic (e.g. F, t, r) with confidence intervals, effect sizes, degrees of freedom and P value noted<br>*Give P values as exact values whenever suitable.*
☐	☒	For Bayesian analysis, information on the choice of priors and Markov chain Monte Carlo settings
☐	☒	For hierarchical and complex designs, identification of the appropriate level for tests and full reporting of outcomes
☒	☐	Estimates of effect sizes (e.g. Cohen's d, Pearson's r), indicating how they were calculated

Our web collection on statistics for biologists contains articles on many of the points above.

Software and code

Policy information about availability of computer code

Data collection	n/a
Data analysis	Statistical code for all analyses and figures is available at: https://github.com/gabrielsaffa/african_mole_rats.

For manuscripts utilizing custom algorithms or software that are central to the research but not yet described in published literature, software must be made available to editors and reviewers. We strongly encourage code deposition in a community repository (e.g. GitHub). See the Nature Portfolio guidelines for submitting code & software for further information.

Data

Policy information about availability of data

All manuscripts must include a data availability statement. This statement should provide the following information, where applicable:

- Accession codes, unique identifiers, or web links for publicly available datasets
- A description of any restrictions on data availability
- For clinical datasets or third party data, please ensure that the statement adheres to our policy

All data generated or analysed during this study are included in this published article (and its supplementary information files).

Field-specific reporting

Please select the one below that is the best fit for your research. If you are not sure, read the appropriate sections before making your selection.

☒ Life sciences ☐ Behavioural & social sciences ☐ Ecological, evolutionary & environmental sciences

For a reference copy of the document with all sections, see nature.com/documents/nr-reporting-summary-flat.pdf

Life sciences study design

All studies must disclose on these points even when the disclosure is negative.

Sample size	In order to have a representative sample of the adult population a minimum of 30 individuals per species were selected (except for Bathyergus janetta, which is a rare and scarcely-known species not available in museum collections). The selection of individuals also intended to represent the initial postnatal development of the species, including newborns, pups, and juveniles. No calculation of sample sizes were performed for selection of early ontogentic stages. Nonetheles, the ontogenetic sequences analyzed are sufficient for the determination of the intial and end-points of postnatal development for each species. For example, the inclusion of B. janetta intends to contribute to our knowledge of the initial development of the genus Bathyergus.
Data exclusions	N/A
Replication	N/A
Randomization	Adult individuals were selected randomly to represent the whole variation within the species. This selection included individuals of both sexes.
Blinding	N/A

Reporting for specific materials, systems and methods

We require information from authors about some types of materials, experimental systems and methods used in many studies. Here, indicate whether each material, system or method listed is relevant to your study. If you are not sure if a list item applies to your research, read the appropriate section before selecting a response.

Materials & experimental systems

n/a	Involved in the study
☒	☐ Antibodies
☒	☐ Eukaryotic cell lines
☒	☐ Palaeontology and archaeology
☐	☒ Animals and other organisms
☒	☐ Human research participants
☒	☐ Clinical data
☒	☐ Dual use research of concern

Methods

n/a	Involved in the study
☒	☐ ChIP-seq
☒	☐ Flow cytometry
☒	☐ MRI-based neuroimaging

Animals and other organisms

Policy information about studies involving animals; ARRIVE guidelines recommended for reporting animal research

Laboratory animals	Specimens from captivity: Heterocephalus glaber and Fukomys damarensis
Wild animals	N/A
Field-collected samples	Most specimens come from field collected samples, from different institutional efforts in different periods. Details for capture, maintenance, handling and permits are presented in previous research (see Montoya-Sanhueza & Chinsamy (2017) and Montoya-Sanhueza et al., (2021)). Table S1 summarize the most important information for the origin of samples, including the following institutions: Kalahari Mole-Rat Project, Kalahari Research Centre (KRC); Department of Biological Sciences, University of Cape Town (UCT); Mammal Research Institute, Department of Zoology and Entomology, University of Pretoria (UP); Department of Zoology, Faculty of Science, University of South Bohemia (USB); Paläontologisches Institut und Museum, Universität Zürich (UZ).<br><br>-Montoya-Sanhueza, G, Bennett, NC, Oosthuizen, MK, Dengler-Crish, CM, Chinsamy, A. 2021. Long bone histomorphogenesis of the naked mole-rat: histodiversity and intraspecific variation. J Anat.: 1– 25.<br><br>-Montoya-Sanhueza, G & A. Chinsamy. 2017. Long bone histology of the subterranean rodent Bathyergus suillus (Bathyergidae): ontogenetic pattern of cortical bone thickening. Journal of Anatomy, 230:203-233.

Ethics oversight	See above

Note that full information on the approval of the study protocol must also be provided in the manuscript.
